# Comparative short-term efficacy and acceptability of a combination of pharmacotherapy and psychotherapy for depressive disorder in children and adolescents: a systematic review and meta-analysis

**DOI:** 10.1186/s12888-022-03760-2

**Published:** 2022-02-22

**Authors:** Yajie Xiang, Pim Cuijpers, Teng Teng, Xuemei Li, Li Fan, Xueer Liu, Yuanliang Jiang, Kang Du, Jingyuan Lin, Xinyu Zhou, Peng Xie

**Affiliations:** 1 Department of Neurology, the First Affiliated Hospital of Chongqing Medical University, 1 Youyi Road, Yuzhong District, Chongqing, 400016 China; 2 NHC Key Laboratory of Diagnosis and Treatment on Brain Functional Diseases, the First Affiliated Hospital of Chongqing Medical University, Chongqing, China; 3 Chongqing Key Laboratory of Neurobiology, Chongqing, China; 4 Department of Clinical, Neuro and Developmental Psychology, Amsterdam Public Health Research Institute, Vrije Universiteit, Amsterdam, The Netherlands; 5 School of Psychology, Shenzhen University, Shenzhen, China; 6 Department of Psychiatry, the First Affiliated Hospital of Chongqing Medical University, 1 Youyi Road, Yuzhong District, Chongqing, 400016 China

**Keywords:** Depressive disorder, Children and adolescents, Combined therapy, Efficacy, Meta-analysis

## Abstract

**Background:**

Although the clinical efficacy and safety of combination of pharmacotherapy and psychotherapy in the treatment of depressive disorders in children and adolescents have been studied, the results remain controversial. This meta-analysis aimed to study the short-term efficacy and acceptability of combined therapy for children and adolescents with depressive disorders.

**Methods:**

We conducted a systematic search in multiple databases for randomised controlled trials (RCTs), up to 31 December 2020, that assessed the combination of pharmacotherapy and psychotherapy against other active treatment options (pharmacotherapy, psychotherapy and placebo combined psychotherapy) in children and adolescents ( ≤ 18 years old) with depressive disorder. This study was registered with PROSPERO (CRD42020196701).

**Results:**

A total of 14 RCTs involving 1,325 patients were included. For the primary and secondary outcomes, there were no statistically significant differences between the compared interventions in terms of remission (odds ratios [OR] = 1.37; 95% confidence interval [CI]: 0.93 to 2.04), acceptability (OR = 0.99; 95% CI: 0.72 to 1.38), efficacy (standardised mean differences = -0.07; 95% CI: -0.32 to 0.19), and suicidality (OR = 1.17; 95% CI: 0.67 to 2.06). Limited evidence showed that the combination of fluoxetine (OR = 1.90, 95% CI: 1.10 to 3.29) or non-selective serotonin reuptake inhibitors (non-SSRI) (OR = 2.46, 95% CI: 1.06 to 5.72) with cognitive-behavioural therapy (CBT) was superior to other active treatment options. Most included trials were rated as ‘some concerns’ in terms of risk of bias assessment.

**Conclusion:**

There is no evidence from the limited available data that all combined therapies are superior to other active treatment options for the acute treatment of depressive disorder in children and adolescents. However, it showed that fluoxetine or non-SSRI pharmacotherapies combined with CBT might be superior to other therapies in short-term. Mixed characteristics (e.g. age) and small sample size of non-SSRI combined therapy may influence the generalisability of the results.

**Supplementary Information:**

The online version contains supplementary material available at 10.1186/s12888-022-03760-2.

## Introduction

The observed prevalence of depression is estimated to be 3.2% in children and adolescents in the United States [1]. Depression causes extensive morbidity and mortality in children and adolescents, and depression-related suicide is the third leading cause of death among them [2]. Compared with adults, depressed children and adolescents are more often misdiagnosed and have more frequent suicide ideation and attempts [3]. Depressive disorder in children and adolescents is debilitating and affects family, psychosocial and academic functions [4].

Several kinds of interventions have been used to treat children and adolescents with depression, from pharmacotherapy to psychotherapy. Psychotherapies, especially cognitive-behavioural therapy (CBT) and interpersonal psychotherapy (IPT) are demonstrated to be effective [5]. As for pharmacotherapy, fluoxetine is significantly more efficacious than placebo, whereas some other antidepressants may produce an increased risk of suicidality [6]. Previous studies have shown that antidepressants have minor but significant contributions to the overall efficacy of the combined treatment of depression in adults [7]. However, whether the combination of pharmacotherapy and psychotherapy is more beneficial than pharmacotherapy or psychotherapy alone remains unclear in children and adolescents [8]. Based on our previous findings, fluoxetine combined with CBT was superior to CBT alone but not more effective than fluoxetine alone [9]. The previous network meta-analysis integrated direct evidence with indirect evidence, which indicated considerable heterogeneity and inconsistency, and most results were assessed as having low confidence in the evidence. Therefore, whether the combination of pharmacotherapy and psychological intervention is more efficacious than other active treatment options remain controversial. This meta-analysis aimed to integrate direct evidence and examine the acute treatment phase potential benefits, acceptability, and harms of a combination of pharmacotherapy and psychotherapy for children and adolescents with depressive disorders.

## Methods

### Search strategy, selection criteria, and risk of bias

The protocol was registered with PROSPERO (CRD42020196701). We updated the literature search of our previous publications [5, 6, 9] to identify trials of the combination of pharmacotherapy and psychotherapy. Further, we searched for eligible published and unpublished randomised controlled trials (RCTs) on PubMed, Embase, CENTRAL, PsycINFO, Web of Science, CINAHL, LiLACS, ProQuest Dissertations, and international trial registers from inception until 31 December 2020 (see details in Appendix 1). Additionally, we searched drug approval agencies websites (such as the FDA), conference proceedings, relevant scientific journals, and related articles or reviews. We contacted the authors to provide incomplete data on the published studies or original data for unpublished studies. Eligible studies included a combination of pharmacotherapy and psychotherapy compared with other active treatment options (pharmacotherapy alone, psychotherapy alone or pill placebo plus psychotherapy) for the treatment of children and adolescents (younger than 18 years old) with a primary diagnosis of depressive disorder (including major depressive disorder [MDD], dysthymia, and other specified types) based on standardised diagnostic criteria (such as Diagnostic and Statistical Manual of Mental Disorders and International Classification of Diseases). Trials involved patients with comorbid mental disorders (e.g. anxiety disorder and attention deficit hyperactivity disorder) were also eligible. However, trials assessing participants with psychotic depression or treatment-resistant depression were excluded, mainly because their treatment responses were different [10]. No restriction was put on language.

All oral medications or alternative treatments within the therapeutic dose range were considered to be eligible. It was considered as a well-structured intervention when psychotherapy was based on a clear manual for therapists or participants. The arm of pill placebo alone or psychotherapy control conditions alone (treatment as usual, no treatment, and waitlist) only were not examined in the meta-analysis. The acute treatment phase was defined as 4-16 weeks. Trials with less than four weeks of treatment were excluded because the benefits of most antidepressants usually begin at least four weeks after commencement. If a study reported data at multiple time points within a predetermined acute phase range or more than 16 weeks, we used eight weeks (or the nearest to eight weeks). The double-blind design is challenging to perform in a psychotherapy trial or a combined trial of psychotherapy [11], therefore the design of blind was unrestricted. Trials in which participants were assessed using physician/parent-rated or self-rated depression scales were all eligible [12]. To reduce heterogeneity between trials, trials with quasi-randomised design and those with a total sample size of less than 10 were excluded.

Two researchers (Y.X. and T.T.) screened the studies, extracted data, and assessed the risk of bias, respectively. Data were extracted using a standardised data collection form. Any discrepancies in data extraction and quality assessment were resolved by consensus and arbitration by senior investigators (X.Z. and P.X.). The risk of bias was assessed using the Cochrane Collaboration’s risk of bias version 2.0 (ROB 2.0) tool [13]. Studies were evaluated in the following six domains: (1) bias arising from the randomisation process; (2) bias due to deviations from intended interventions; (3) bias due to missing outcome data; (4) bias in the measurement of the outcome; (5) bias in the selection of the reported result; and (6) overall bias. Each domain was rated as ‘low risk’, ‘some concerns’, or ‘high risk’.

### Outcomes

The primary outcomes included (1) remission (as a dichotomous data), estimated by the total number of patients who met the criteria for remission, was defined as a depressive symptom score below the threshold (e.g. < 28 for Children’s Depression Rating Scale-Revised [CDRS-R]) in the trials; and (2) acceptability, defined as all-cause discontinuation, was measured by the percentage of participants who withdrew from the study for any reason before the end of treatment. Secondary outcomes included (1) efficacy at post-treatment (as continuous data), estimated by the overall change score of the depressive symptom scales (e.g. CDRS-R or Hamilton Rating Scale for Depression scale) completed by self, parents, or clinicians from baseline to the end of treatment; and (2) suicidality, measured by the number of patients reporting suicidal ideation or suicidal attempt/behaviour in the treatment phase.

Where multiple depressive symptom scales were reported in a study, we extracted data from a predefined hierarchy based on psychometric properties and appropriateness for children and adolescents (see details in Appendix 2). Moreover, in a study where different raters reported the scale, we preferred the parent/clinician- rated outcomes to the self-rated ones [14].

### Data analysis

We calculated effect sizes and pooled estimates of effect across studies with a random-effects model using the RevMan 5 software (Cochrane Information Management System) due to considerable clinical heterogeneity among trials. We summarised the standardised mean differences (SMD) for continuous data and odds ratios (ORs) for dichotomous data, as well as 95% confidence intervals (CIs). Heterogeneity was assessed using Q and I^2^ statistics. Statistical significance was set at *p* < 0.05, and values of 25%, 50%, and 75% displayed low, moderate, and substantial heterogeneity, respectively. Power and Precision (version 4.0) was used to carry out power calculations. When the mean values or standard deviations (SDs) of continuous outcomes were missing, we calculated values by converting the *p* values, t-values, CIs, or standard errors.

Considering that effectiveness varies depending on the type of medication and psychotherapy used, we conducted three subgroup analyses, these were according to the types of combined pharmacotherapies, types of combined psychotherapies, and types of active treatment options (pharmacotherapy, psychotherapy and placebo combined psychotherapy). We also conducted subgroup analyses regarding (1) mean baseline severity of the depressive disorder (mild vs. moderate to serve, thresholds presented in Appendix 3); (2) treatment duration (treatment ≤ 8 weeks vs. treatment > 8 weeks); (3) conducted country (the USA vs. non-USA); and (4) risk of bias (low risk vs. some concerns vs. high risk). For continuous variables, we performed meta-regression analyses according to the following variables: publication year, mean age, and percentage of female patients. We also conducted sensitivity analyses by excluding some trials from the fundamental analysis for the non-blind design trials, potential publication bias trials, and high-risk trials of ROB 2.0. Potential publication bias was assessed using inverted funnel plots and Egger’s test. The protocol followed the PRISMA recommendations for meta-analysis [15]. All tests were two-sided, and *p* values less than 0.05 were considered statistically significant.

## Results

### Selection, inclusion, and characteristics of studies

**Fig. 1:**
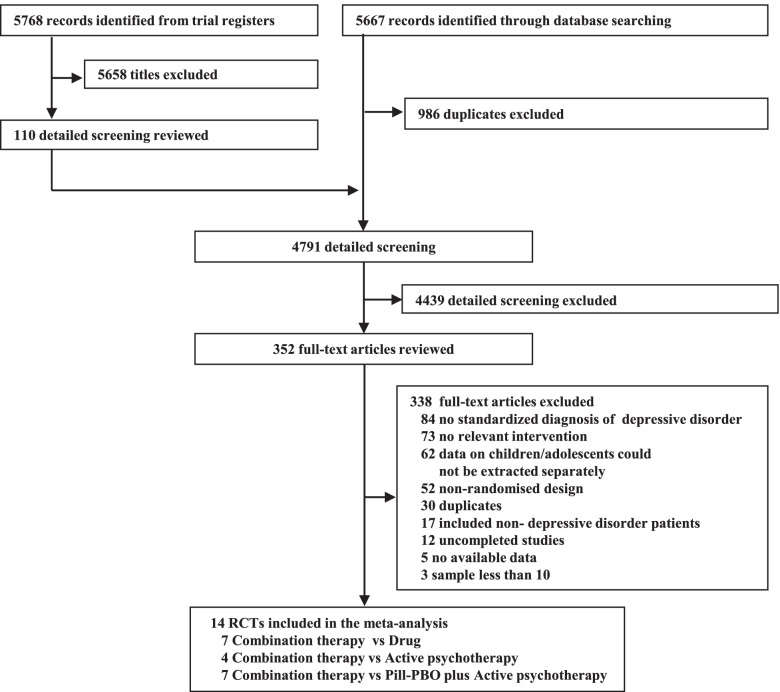
Flow chart of study selection. Pill-PBO= pill placebo

Overall, we searched 11,435 citations, identified the full text of 352 possibly eligible studies, and finally included 14 RCTs (1,325 participants, Fig. 1) published from 1997 to 2020. Six pharmacotherapies (imipramine, fluoxetine, sertraline, omega-3, bupropion, and venlafaxine), three different psychotherapies (CBT, IPT, and psychoeducational psychotherapy [PEP]), and the pill placebo plus psychotherapy were assessed. A total of 567 participants were randomly assigned to combined therapy, 401 to pharmacotherapy alone, 167 to psychotherapy alone, and 190 to pill placebo plus psychotherapy.

The clinical and methodological characteristics of the included studies are shown in Table 1. A total of 13 trials recruited only patients with MDD [8, 16–27], and one recruited patients with MDD, dysthymia, and depressive disorder-not otherwise specified [28]. In 10 of the included studies, pharmacotherapies involved selective serotonin reuptake inhibitors (SSRIs), with five studies of fluoxetine, three of sertraline, and two of mixed SSRIs. Additionally, the other four studies included imipramine, venlafaxine, omega-3, and bupropion. Psychotherapy involved CBT in 12 studies, IPT in one, and PEP in one. Seven studies compared combination therapies with pharmacotherapies alone, four trials compared combination therapies with psychotherapies alone, and seven studies compared combination therapies with placebo plus psychological therapies. The average study sample size was 95, ranging from 10 to 327. One trial (7.1%) enrolled only children, 11 (78.6%) enrolled only adolescents, and the remaining two (14.3%) enrolled both children and adolescents. The mean age ranged from 7 to 18 years (mean, 15.2 years old; SD, 2.0), and 753 (56.8%) of the participants were female. Four (28.6%) studies had a multiple-arm design. Nine (64.3%) studies were conducted in the USA and five (35.7%) in other countries, including Australia, Romania, South Korea, and the United Kingdom. The median treatment duration was 12 weeks (interquartile range [IQR], 8–12). Eight trials had a double-blind design; three trials had a single-blinding design, that is, the assessors were blinded to the interventions of patients; and one trial had a non-blinding design with a self-reported scale assessment. There was insufficient information to identify blinding of outcome assessment in two trials also using self-reported scale assessment.

**Table 1:** Randomised controlled trials included in the systematic review and meta-analysis

Trial	Diagnostic criteria	Type of depression	Treatments (dose range/number of sessions)	No. randomised	Treatment duration (selected time-point, weeks)	Age range and mean age (years)	Proportion of female	Recruitment	Baseline severity scale	Mean baseline severity, mean (SD)	Type of blinding
Bernstein 2000	DSM-III-R	MDD	Imipramine+CBT (3 mg/day per kg; 8 sessions) Pill-PBO+CBT (8 sessions)	31/32	8 (8)	12-18 (13.9)	60%	United States	CDRS-R (clinician-reported)	49.7 (10.5)	Double-blind
Clarke 2005	DSM-IV	MDD	SSRI+CBT (unfixed dose; 5.3 sessions) SSRI (10-60mg/day)	77/75	12 (12)	12-18 (15.3)	78%	United States	HAMD-14	21.5 (6.3)	Single-blind (assessor-blind)
Cornelius 2009	DSM-IV	MDD	Fluoxetine+CBT (10-20 mg/day; 9 sessions) Pill-PBO+CBT (9 sessions)	24/26	12 (12)	15-20 (Not stated)	56%	United States	HAMD-27 (clinician-reported)	20.0 (8.5)	Double-blind
Davey 2019	DSM-IV	MDD	Fluoxetine+CBT (20-40 mg/day; 12 sessions) Pill-PBO+CBT (12 sessions)	24/25	12 (12)	15-25 (19.6 )^a^	67%	Australia	MADRS (clinician-reported)	32.0 (5.5)	Double-blind
Deas 2000	DSM-IV	MDD	Sertraline+CBT (25-100mg/day; 12 sessions) Pill-PBO+CBT (12 sessions)	5/5	12 (12)	15-18 (16.6)	20%	United States	HAMD-24 (clinician-reported)	20.6 (5.2)	Double-blind
Fristad 2019	DSM-IV-TR	MDD	Omega-3+PEP (1870 mg/day; 12 sessions) Pill-PBO+PEP (12 sessions) Omega-3 (1870 mg/day)	17/19/18	12 (12)	7-14 (11.6)	43%	United States	CDRS-R (clinician-reported)	41.4 (10.2)	Double-blind
Goodyer 2008	DSM-IV	MDD	SSRI +CBT (unfixed dose; 12 sessions) SSRI (unfixed dose)	105/103	12 (12)	11-17 (14.0)	74%	United Kingdom	CDRS-R (clinician-reported)	58.9 (9.9)	Single-blind (assessor-blind)
Gunlicks-Stoessel 2019	DSM-IV-TR	MDD	Fluoxetine+IPT (10-20mg/day; 12 sessions) IPT (12 sessions )	7/6	8 (8)	12-18 (14.8)	77%	United States	CDRS-R (clinician-reported)	55.3 (10.5)	Single-blind (assessor-blind)
Iftene 2015	DSM-IV	MDD	Sertraline+CBT (25-50mg/day; 16 sessions) Sertraline (25-50mg/day) CBT (16 sessions)	27/33/28	16 (8)	11-17 (15.3)	56%	Romania	CDI (self-reported)	24.0 (5.8)	Not stated (self-reported scale)
Kim 2012	DSM-IV	MDD	Buproion+CBT (150-300mg/day; 8 sessions) Buproion (150-300mg/day)	35/37	8 (8)	13-18 (16.1)	0%	South Korea	BDI (self-reported)	33.0 (8.7)	Not stated (self-reported scale)
Mandoki 1997	DSM-IV	MDD	Venlafaxine+CBT (12.5-75 mg/day; 6 sessions) Pill-PBO+CBT (6 sessions)	20/20	6 (6)	8-17 (12.8)	76%	United States	CDRS (clinician-reported)	34.8 (Not stated)	Double-blind
March 2004	DSM-IV	MDD	Fluoxetine+CBT (10-40 mg/day; 15 sessions) Fluoxetine (10-40 mg/day) CBT (15 sessions)	107/109/111	12 (12)	12-17 (14.6)	54%	United States	CDRS-R (clinician-reported)	59.8 (4.5)	Double-blind (fluoxetine); assessor-blind (CBT, fluoxetine+CBT)
Melvin 2006	DSM-IV	MDD, dysthymia, or DDNOS	Sertraline + CBT (25-100 mg/day; 12 sessions) Sertraline (25-100 mg/day per kg) CBT (12 sessions)	25/26/22	12 (12)	12-18 (15.3)	66%	Australia	RADS (self-reported)	84.2 (13.2)	Non-blind (self-reported scale)
Riggs 2007	DSM-IV	MDD	Fluoxetine+CBT (20 mg/day; 16 sessions) Pill-PBO+CBT (16 sessions)	63/63	16 (8)	13-19 (17.2)	33%	United States	CDRS-R (clinician-reported)	56.8 (13.4)	Double-blind

^a^Only patients younger than 18 years old were included in this study

*SD* Standard deviation; *DSM* Diagnostic and Statistical Manual of Mental Disorders; *MDD* Major depressive disorder; *CBT* Cognitive behavioural therapy; *Pill-PBO* Pill placebo; *CDRS-R* Children’s Depression Rating Scale-Revised; *SSRI* Selective serotonin reuptake inhibitor; *HAMD* Hamilton Rating Scale for Depression; *MADRS* Montgomery-Asberg Depression Rating Scale; *PEP* Psychoeducational psychotherapy; *IPT* Interpersonal therapy; *CDI* Children’s Depression Inventory; *BDI* Beck Depression Inventory; *DDNOS* Depressive disorder-not otherwise specified; *RADS* Reynolds Adolescent Depression Scale

### Meta-analysis results for primary outcomes

**Fig. 2:**
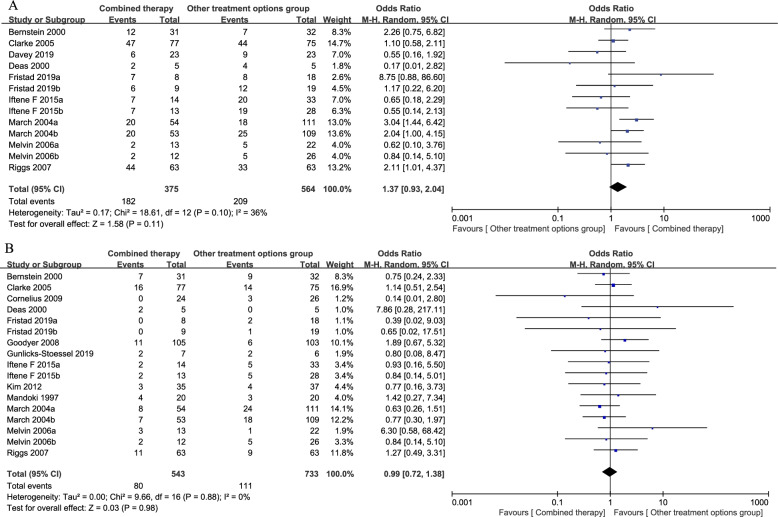
Forest plots of meta-analysis for primary outcomes. **A**. Remission; **B**. Acceptability.

The results of the meta-analysis for all outcomes are presented in Table 2. Nine studies (939 patients) presented remission data compared combined therapies with pharmacotherapies or psychotherapies alone. The remission did not change substantially with the pooled OR of 1.37 (95% CI = 0.93 to 2.04, *p* = 0.11) from the random-effects model, and a moderate significant heterogeneity (*p* = 0.10, I^2^ = 36%, Fig. 2A) was observed. The statistical power of the effect size was 93%. Thirteen studies (1,276 patients) reported all-cause discontinuation with compared combination therapies with other active treatment options group; the two kinds of interventions did not materially differ, with a pooled OR of 0.99 (95% CI = 0.72 to 1.38, *p* = 0.98) and no heterogeneity among studies (*p* = 0.88, I^2^ = 0%, Fig. 2B). The statistical power of the effect size was 5%.

**Table 2:** Results of all outcomes (individual studies and overall effect)

Studies	Remission	Acceptability	Efficacy at post treatment	Suicidality
	Odds ratio (95% CI)	Odds ratio (95% CI)	Standard mean difference (95% CI)	Odds ratio (95% CI)
Bernstein 2000	2.26 (0.75 to 6.82)	0.75 (0.24 to 2.33)	-0.44 (-0.94 to 0.06)	Not reported
Clarke 2005	1.10 (0.58 to 2.11)	1.14 (0.51 to 2.54)	0.08 (-0.28 to 0.43)	Not reported
Cornelius 2009	Not reported	0.14 (0.01 to 2.80)	0.27 (-0.29 to 0.83)	Not estimable
Davey 2019	0.55 (0.16 to 1.92)	Not reported	0.10 (-0.46 to 0.66)	1.82 (0.57 to 5.79)
Deas 2000	0.17 (0.22 to 6.20)	7.86 (0.28 to 217.11)	0.36 (-0.90 to 1.61)	Not reported
Fristad 2019a	8.75 (0.88 to 86.60)	0.39 (0.02 to 9.03)	0.09 (-0.76 to 0.94)	Not reported
Fristad 2019b	1.17 (0.22 to 6.20)	0.65 (0.02 to 17.51)	0.00 (-0.80 to 0.80)	Not reported
Goodyer 2008	Not reported	1.89 (0.67 to 5.32)	0.19 (-0.09 to 0.47)	0.85 (0.30 to 2.43)
Gunlicks-Stoessel 2019	Not reported	0.80 (0.08 to 8.47)	0.24 (-0.86 to 1.33)	Not estimable
Iftene F 2015a	0.65 (0.18 to 2.29)	0.93 (0.16 to 5.50)	0.38 (-0.25 to 1.01)	Not estimable
Iftene F 2015b	0.55 (0.14 to 2.13)	0.84 (0.14 to 5.01)	0.11 (-0.54 to 0.77)	Not estimable
Kim 2012	Not reported	0.77 (0.16 to 3.73)	-0.53 (-1.02 to -0.03)	Not reported
Mandoki 1997	Not reported	1.42 (0.27 to 7.34)	0.73 (0.09 to 1.38)	Not reported
March 2004a	3.04 (1.44 to 6.42)	0.63 (0.26 to 1.51)	-1.22 (-1.57 to -0.87)	1.25 (0.29 to 5.42)
March 2004b	2.04 (1.00 to 4.15)	0.77 (0.30 to 1.97)	-0.61 (-0.95 to -0.28)	0.67 (0.17 to 2.57)
Melvin 2006a	0.62 (0.10 to 3.76)	6.30 (0.58 to 68.42)	0.34 (-0.35 to 1.03)	5.40 (0.20 to 142.71)
Melvin 2006b	0.84 (0.14 to 5.10)	0.84 (0.14 to 5.10)	-0.02 (-0.70 to 0.66)	0.50 (0.05 to 5.03)
Riggs 2007	2.11 (1.01 to 4.37)	1.27 (0.49 to 3.31)	-0.33 (-0.68 to 0.02)	4.20 (0.46 to 38.71)
Total				
**Overall effect**	1.37 (0.93 to 2.04)	0.99 (0.72 to 1.38)	-0.07 (-0.32 to 0.19)	1.17 (0.67 to 2.06)
***P*** **for overall effect**	0.11	0.98	0.60	0.58
**I**^**2**^	36%	0%	76%	0%
***P*** **for heterogeneity**	0.10	0.88	< 0.00001	0.65
**Statistical power**	93%	5%	6%	16%

*CI* confidence interval; *P* level of significance; I^2^ measure of heterogeneity

### Meta-analysis results for secondary outcomes

A total of 14 studies (1,276 patients) reported efficacy at short-term post-treatment compared combination therapies with other active treatment options. The overall changes in the score of the depressive symptom scales were not associated with a significant decrease, and the pooled SMD was -0.07 (95% CI = -0.32 to 0.19, *p* = 0.60) between groups with high and significant heterogeneity (*p* <0.00001, I^2^ = 76%, Table 2) among studies. The statistical power of the effect size was 6%. In terms of suicidality, all eight studies (932 patients) presented data on suicidality in combined therapy and other active treatment options. Further, there was no statistically significant effect (OR = 1.17, 95% CI = 0.67 to 2.06, *p* = 0.58) and no heterogeneity (*p* = 0.65, I^2^ = 0%, Table 2). The statistical power of the effect size was 16%.

### Subgroup and sensitivity analyses

Owing to significant heterogeneity in remission, various subgroup analyses were conducted to explore potential bias from the type of intervention (Table 3). The combination of fluoxetine (OR = 1.90, 95% CI = 1.10 to 3.29) or non-SSRI pharmacotherapies (OR = 2.46, 95% CI = 1.06 to 5.72) with psychotherapies were superior to other active treatment options; however, CBT was the only psychotherapy available for analysis here. The subgroup analyses in terms of the type of combined psychotherapies and the type of other active treatment options showed no significant difference between interventions (combination therapies vs. other active treatment options), but with significant heterogeneity among studies. Furthermore, we performed subgroup analyses regarding some main baseline characteristics, including mean baseline severity of the depressive disorder, treatment duration, conducted countries, and risk of bias. The results showed that combined therapy was more efficacious than other active treatment options in studies conducted in the USA (OR = 1.90, 95% CI = 1.33 to 2.73) and studies with a ‘high risk’ of bias (OR = 1.89, 95% CI = 1.19 to 3.01). The above subgroup analyses did not show any significant results for acceptability (Appendix 4). Meta-regression analyses showed that none of the continuous modifiers (publication year, mean age, percentage of female patients, and treatment duration) was associated with remission rate or acceptability in the combined treatment compared with other active treatment options (all *p* > 0.05). Sensitivity analyses for non-blind design studies, potential publication bias trials, and high-risk trials of ROB 2.0 showed that there was no substantial change compared with primary analysis (Appendix 5).

**Table 3:** Subgroup analysis of remission at post-treatment for all studies

Variable	No of studies	Odds ratio (95% CI)	Chi^**2**^	I^**2**^	***P*** value	Statistical power
**Type of other active treatment options**						
Combined therapy vs pharmacotherapy	5	1.36 (0.83 to 2.23)	4.80	17%	0.31	62%
Combined therapy vs psychotherapy	3	1.18 (0.33 to 4.23)	6.18	68%	0.05	36%
Combined therapy vs Pill-PBO combined psychotherapy	5	1.36 (0.67 to 2.79)	6.17	35%	0.19	27%
**Type of medication combined therapy**						
Fluoxetine combined therapy	4	**1.90 (1.10 to 3.29)**	5.32	44%	0.15	99%
Other SSRIs combined therapy	6	0.83 (0.51 to 1.35)	2.58	0%	0.77	6%
Non-SSRIs combined therapy	3	**2.46 (1.06 to 5.72)**	0.54	0%	0.77	28%
**Type of psychotherapy combined therapy**						
CBT combined therapy	11	1.31 (0.87 to 1.98)	16.23	38%	0.09	92%
Non-CBT combined therapy	2	2.79 (0.76 to 10.22)	0.48	0%	0.49	36%
**Severity of baseline symptom**						
Mild severity	3	1.45 (0.28 to 7.46)	3.63	45%	0.16	14%
Moderate to server severity	10	1.39 (0.93 to 2.06)	13.94	35%	0.12	94%
**Treatment duration**						
Treatment ≤ 8 weeks	4	1.31 (0.65 to 2.64)	5.14	42%	0.16	23%
Treatment > 8 weeks	9	1.42 (0.87 to 2.33)	12.45	36%	0.13	88%
**Country**						
USA	8	**1.90 (1.33 to 2.73)**	8.07	13%	0.33	100%
Non-USA	5	0.62 (0.32 to 1.17)	0.18	0%	1.00	40%
**Risk of bias**						
Low risk	3	1.39 (0.40 to 4.86)	3.59	44%	0.17	5%
Some concerns	6	0.92 (0.47 to 1.79)	6.89	27%	0.23	12%
High risk	4	**1.89 (1.19 to 3.01)**	4.32	31%	0.23	98%

Significant results are bolded and underscored

*CI* confidence interval, *P* level of significance of heterogeneity, *I*^*2*^ measure of heterogeneity, *Pill-PBO* Pill placebo, *SSRIs* Selective serotonin reuptake inhibitors, *CBT* Cognitive behavioural therapy, *USA* United States of America

### Quality assessment and publication bias

In terms of study quality according to ROB 2.0, 3 (21.4%) out of 14 trials were rated as having a high risk of bias, 8 (57.1%) as having some concerns, and 3 (21.4%) as having a low risk of bias (Appendix 6). The funnel plot and Egger’s tests did not suggest a significant publication bias for all outcomes (all *p* > 0.05, Appendix 7).

## Discussion

This meta-analysis was based on 14 RCTs including 1,325 children and adolescents with depressive disorder randomly assigned to the combination therapy or other active treatment options. To the best of our knowledge, this is the most comprehensive synthesis to assess combination therapies for depressive disorders in children and adolescents in a meta-analysis. There is no evidence from the limited available data that all combined therapies are superior to other active treatment options for the acute treatment of depressive disorder in children and adolescents. However, it showed that fluoxetine or non-SSRI pharmacotherapies combined with CBT might be superior to other therapies in the short-term. Mixed characteristics (e.g. age) and small sample size of non-SSRI combined therapy may reduce the generalisability of the results.

Our findings were similar to those in previous studies involving children and adolescents [29–31] as well as adults [32]. However, those reviews also differed from ours in some aspects. One study only analysed CBT combined with newer-generation antidepressants therapies [29], one study included trials with ‘treatment resistant’ participants [30], and another included only patients with MDD [31]. Given the limitations of the data, the authors of these studies cautioned against drawing firm conclusions. The heterogeneity among studies also limited our conclusion, which may be because of the different types of pharmacotherapies and psychotherapies. Further subgroup analysis of types of medications showed that fluoxetine combined with CBT was more efficacious than other active treatment options in terms of remission at post-treatment. It is noticed that, CBT was the only psychotherapy available in these studies. Fluoxetine is one of the most widely studied SSRIs, and CBT is one of the most commonly studied psychotherapies; both of them have shown some effectiveness in the treatment of depressive disorder in children and adolescents [31]. The efficacy of fluoxetine combined with CBT compared with CBT alone has also been demonstrated in our previous network meta-analysis, which integrates direct and indirect evidence [9]. Additionally, the non-SSRIs included in the present study are imipramine [16] and Omega-3 [20]; however, they were only reported in one study each, which may be prone to bias of results. Therefore, limited data may reduce the generalisability of the results of non-SSRI combined therapy. Further, most of these pooled effect sizes were small to medium with some uncertainty, which could be due to the relatively small sample size and wide confidence intervals. Thus, caution should be exercised when explaining the statistical superiority in this study.

It was observed that the dropout prevalence was approximately 23% (ranging from 20% to 27%) among depressed adolescents treated with antidepressants [33]. The most critical factor related to the high rate of dropout was the side effects of medications (such as suicide-related events, mania, skin rash, and headaches) [33]. It has been demonstrated that the optimal dropout rate should not exceed 20% because exceeding this number could indicate questionable representativeness, reliability, and generalisability [34, 35]. No significant difference was observed between all the compared groups with low statistical power or in the subgroup analyses of acceptability. In terms of the included studies, five (35.7%) clinical trials [16, 17, 22, 26] reported dropout rates of more than 20%. Among them, all analysed interventions included pharmacotherapies, psychotherapies, and their combination; two studies [19, 22] had small sample sizes of less than 10 in each intervention group. All of the above five studies included patients older than 12 years. It was previously reported that compared with adults who received pharmacotherapy alone, adults receiving a combination of pharmacotherapy and psychotherapy had a lower dropout rate [36]. Concerning age, it was reported that individuals over the age of 16 had a higher dropout rate than younger patients [37]. However, subgroup analyses of different interventions and the meta-regression of mean age did not show significant results in the present study. Thus, we could not conclude how these potential moderators influence the dropout rate.

Since 2003, the FDA has been stipulating the risk of suicidal thoughts and attempts related to the use of antidepressants for treating children and adolescents with depression in a black box warning [38]. It was reported that patients under the age of 25 treated with antidepressants are more likely to develop suicidal thoughts than older adults [3]. In our analysis, no difference was observed in the rates of suicidal ideation or attempts related to the observed comparisons in children and adolescents with depressive disorder. This result was in line with our previous network meta-analysis [9]. Another study of ours revealed a significantly increased risk for suicide-related outcomes for children and adolescents administered venlafaxine [6]. However, due to the lack of eligible venlafaxine combined therapy data for assessing suicidality in the present study, we could not comprehensively assess the risk of suicide-related outcomes for these related interventions. Moreover, considerable method variability was observed during the data collection and reporting process, making it challenging to extract eligible and homogenous data for meta-analysis. Untreated depressive disorder in children and adolescents is closely related to a high risk of suicide [2], therefore, suicide-related outcomes should be monitored closely regardless of the treatment types.

Our study had several limitations that should be considered when explaining the results. First, relatively few studies have examined the efficacy and acceptability of combined therapy, limiting the comprehensive and systematic assessment of all kinds of psychotherapy and pharmacotherapy combined therapies. Our literature search was as comprehensive as possible, and we also made every effort to search all available unpublished data and contacted authors for additional related data. Although significant publication bias was not observed in the funnel plots and Egger’s test, we cannot eliminate the possibility that some unpublished studies remain missing or that published studies might overvalue the effect size of treatments. Second, substantial heterogeneity was observed in several of the examined comparisons. This may be because of the different characteristics among trials (e.g. various treatment forms, sample size, mean age, sex ratio, disease severity, comorbidity, suicidality or definition of remission across studies). Subgroup and meta-regression analyses of most parameters revealed some potential heterogeneity among the studies. However, the original data were insufficient to perform subgroup analyses or meta-regression for the comorbidity, suicidality and definition of remission. These may limit the generalizability of present results for the real-world clinical populations [39]. Third, children and adolescents with a wide range of 7-18 years old were included in the present study; however, meta-regression analysis of mean age failed to show significant results. Therefore, there was not enough evidence to conclude whether the benefit of each age group could be different. These data should be analysed and contextualised at the individual patient level, without access to individual patient-level data, we cannot be completely confident about the accuracy of information contained in published studies or clinical study reports [40]. Fourth, most included trials were rated as ‘some concerns’ or ‘high risk’ in terms of risk of bias assessment. Subgroup analysis showed that combined therapy was superior to other active treatment options in studies with a ‘high risk’ of bias in terms of remission. Some ‘high risk’ studies did not report the allocation sequence concealed information because it was difficult to conduct a double-blind design in psychotherapy trials, which essentially limited the interpretation of these results. Moreover, sensitivity analyses excluding trials with a ‘high risk’ of bias and with a non-blinded design was conducted, revealing that the results were not substantially different from those of the overall analysis. Fifth, we excluded studies on psychotic depression and treatment-resistant depression, which might overestimate the efficacy of interventions because the most challenging cases were not assessed. Moreover, we could not analyse other outcomes such as the efficacy of long-term follow-up, adverse events, and quality of life, mainly because they were rarely reported in the included studies. These variables are essential for the decision making of clinicians and patients. Sixth, several alternative psychotherapies have not been well studied for depression in young patients; for example, only one study [22] of interpersonal psychotherapy combined with pharmacotherapy met the inclusion criteria of this meta-analysis. Owing to the limited available studies and data of each outcome, many significant outcomes of the meta-analysis were driven by data from the study in March 2004 [26]. Although this study had a large sample size and was well designed, its generalisability was limited because many participants were recruited from advertisements and might not represent patients in clinical practice.

## Conclusions

The findings from the present meta-analysis demonstrated that there was no evidence that all combined therapies are superior to other active treatment options in the acute treatment of depressive disorder in children and adolescents. Limited evidence showed that fluoxetine or non-SSRI pharmacotherapies combined with CBT might be superior to other active treatment options. Mixed characteristics (e.g. age) and small sample size of non-SSRI combined therapy may reduce the generalisability of the results. Interventions need to move beyond a ‘one size fits all’ approach to individualising treatment, and clinicians should consider the importance of each outcome, type of medication, and patient preferences. Combined therapies are understudied in this age group, and further studies focusing on the moderators of efficacy and alternative interventions are needed.

## Supplementary Information

**Additional file 1.**

## Data Availability

The data that support the findings of this study are available from the corresponding author, PX and XZ, upon reasonable request.

## Abbreviations

CBT: Cognitive-Behavioural Therapy
IPT: Interpersonal Psychotherapy
RCTs: Randomised Controlled Trials
MDD: Major Depressive Disorder
ROB: Risk Of Bias version
CDRS-R: Children’s Depression Rating Scale-Revised
SMD: Standardised Mean Differences
ORs: Odds Ratios
CIs: Confidence Intervals
SDs: Standard Deviations
PEP: Psychoeducational Psychotherapy
SSRIs: Selective Serotonin Reuptake Inhibitors
IQR: Interquartile Range

## References

[1] Ghandour RM, Sherman LJ, Vladutiu CJ, Ali MM, Lynch SE, Bitsko RH (2019). Prevalence and Treatment of Depression, Anxiety, and Conduct Problems in US Children. J Pediatr..

[2] Thapar A, Collishaw S, Pine DS, Thapar AK (2012). Depression in adolescence. The Lancet..

[3] Mokdad AH, Forouzanfar MH, Daoud F, Mokdad AA, El Bcheraoui C, Moradi-Lakeh M (2016). Global burden of diseases, injuries, and risk factors for young people's health during 1990–2013: a systematic analysis for the Global Burden of Disease Study 2013. The Lancet..

[4] Gore FM, Bloem PJ, Patton GC, Ferguson J, Joseph V, Coffey C (2011). Global burden of disease in young people aged 10-24 years: a systematic analysis. Lancet..

[5] Zhou X, Hetrick SE, Cuijpers P, Qin B, Barth J, Whittington CJ (2015). Comparative efficacy and acceptability of psychotherapies for depression in children and adolescents: A systematic review and network meta-analysis. World Psychiatry..

[6] Cipriani A, Zhou X, Del Giovane C, Hetrick SE, Qin B, Whittington C (2016). Comparative efficacy and tolerability of antidepressants for major depressive disorder in children and adolescents: a network meta-analysis. Lancet..

[7] Cuijpers P, van Straten A, Hollon SD, Andersson G (2010). The contribution of active medication to combined treatments of psychotherapy and pharmacotherapy for adult depression: a meta-analysis. Acta Psychiatr Scand..

[8] Davey CG, Chanen AM, Hetrick SE, Cotton SM, Ratheesh A, Amminger GP (2019). The addition of fluoxetine to cognitive behavioural therapy for youth depression (YoDA-C): a randomised, double-blind, placebo-controlled, multicentre clinical trial. Lancet Psychiatry..

[9] Zhou X, Teng T, Zhang Y, Del Giovane C, Furukawa TA, Weisz JR (2020). Comparative efficacy and acceptability of antidepressants, psychotherapies, and their combination for acute treatment of children and adolescents with depressive disorder: a systematic review and network meta-analysis. Lancet Psychiatry..

[10] Weissman MM (2009). Teenaged, depressed, and treatment resistant: what predicts self-harm?. Am J Psychiatry..

[11] Del Giovane CCS, Cipriani A. Combining Pharmacological and Nonpharmacological Interventions in Network Meta-analysis in Psychiatry. JAMA Psychiatry. 2019;0574. 10.1001/jamapsychiatry.2019.0574 PMC6583838 30994881

[12] Boutron IGL, Estellat C, Moher D, Hróbjartsson A, Ravaud P (2007). Reporting methods of blinding in randomized trials assessing nonpharmacological treatments. PLoS Med..

[13] Sterne JAC, Savović J, Page MJ, Elbers RG, Blencowe NS, Boutron I (2019). RoB 2: a revised tool for assessing risk of bias in randomised trials. BMJ..

[14] Cuijpers PLJ, Hofmann SG, Andersson G (2010). Self-reported versus clinician-rated symptoms of depression as outcome measures in psychotherapy research on depression: a meta-analysis. Clin Psychol Rev.

[15] Moher D, Liberati A, Tetzlaff J, Altman DG. Preferred reporting items for systematic reviews and meta-analyses: the PRISMA statement. Bmj. 2009;339:b2535. 10.1136/bmj.b2535 PMC2714657 19622551

[16] Bernstein GA, Borchardt CM, Perwien AR, Crosby RD, Kushner MG, Thuras PD (2000). Imipramine plus cognitive-behavioral therapy in the treatment of school refusal. J Am Acad Child Adolesc Psychiatry..

[17] Clarke G, Debar L, Lynch F, Powell J, Gale J, O'Connor E (2005). A randomized effectiveness trial of brief cognitive-behavioral therapy for depressed adolescents receiving antidepressant medication. J Am Acad Child Adolesc Psychiatry..

[18] Cornelius JR, Bukstein OG, Wood DS, Kirisci L, Douaihy A, Clark DB (2009). Double-blind placebo-controlled trial of fluoxetine in adolescents with comorbid major depression and an alcohol use disorder. Addict Behav..

[19] Deas D, Randall CL, Roberts JS, Anton RF (2000). A double-blind, placebo-controlled trial of sertraline in depressed adolescent alcoholics: a pilot study. Hum Psychopharmacol..

[20] Fristad MA, Vesco AT, Young AS, Healy KZ, Nader ES, Gardner W (2019). Pilot Randomized Controlled Trial of Omega-3 and Individual-Family Psychoeducational Psychotherapy for Children and Adolescents With Depression. J Clin Child Adolesc Psychol..

[21] Goodyer IM, Dubicka B, Wilkinson P, Kelvin R, Roberts C, Byford S (2008). A randomised controlled trial of cognitive behaviour therapy in adolescents with major depression treated by selective serotonin reuptake inhibitors. The ADAPT trial. Health Technol Assess..

[22] Gunlicks-Stoessel M, Mufson L, Bernstein G, Westervelt A, Reigstad K, Klimes-Dougan B (2019). Critical Decision Points for Augmenting Interpersonal Psychotherapy for Depressed Adolescents: A Pilot Sequential Multiple Assignment Randomized Trial. J Am Acad Child Adolesc Psychiatry..

[23] Iftene F, Predescu E, Stefan S, David D (2015). Rational-emotive and cognitive-behavior therapy (REBT/CBT) versus pharmacotherapy versus REBT/CBT plus pharmacotherapy in the treatment of major depressive disorder in youth; a randomized clinical trial. Psychiatry Res..

[24] Kim SM, Han DH, Lee YS, Renshaw PF (2012). Combined cognitive behavioral therapy and bupropion for the treatment of problematic on-line game play in adolescents with major depressive disorder. Computers in Human Behavior..

[25] Mandoki MW, Tapia MR, Tapia MA, Sumner GS, Parker JL (1997). Venlafaxine in the treatment of children and adolescents with major depression. Psychopharmacol Bull..

[26] March J, Silva S, Petrycki S, Curry J, Wells K, Fairbank J (2004). Fluoxetine, cognitive-behavioral therapy, and their combination for adolescents with depression: Treatment for Adolescents With Depression Study (TADS) randomized controlled trial. JAMA..

[27] Riggs PD, Mikulich-Gilbertson SK, Davies RD, Lohman M, Klein C, Stover SK (2007). A randomized controlled trial of fluoxetine and cognitive behavioral therapy in adolescents with major depression, behavior problems, and substance use disorders. Arch Pediatr Adolesc Med..

[28] Melvin GA, Tonge BJ, King NJ, Heyne D, Gordon MS, Klimkeit E (2006). A Comparison of Cognitive-Behavioral Therapy, Sertraline, and Their Combination for Adolescent Depression. J Am Academy Child Adolescent Psychiatr.

[29] Dubicka B, Elvins R, Roberts C, Chick G, Wilkinson P, Goodyer IM (2010). Combined treatment with cognitive-behavioural therapy in adolescent depression: meta-analysis. Br J Psychiatry..

[30] Hetrick SE, Cox GR, Merry SN (2011). Treatment-resistant depression in adolescents: is the addition of cognitive behavioral therapy of benefit?. Psychol Res Behav Manag..

[31] Cox GR, Callahan P, Churchill R, Hunot V, Merry SN, Parker AG, et al. Psychological therapies versus antidepressant medication, alone and in combination for depression in children and adolescents. Cochrane Database Syst Rev. 2014;(11):CD008324. 10.1002/14651858.CD008324.pub3 PMC8556660 25433518

[32] von Wolff A, Hölzel LP, Westphal A, Härter M, Kriston L (2012). Combination of pharmacotherapy and psychotherapy in the treatment of chronic depression: a systematic review and meta-analysis. BMC Psychiatry..

[33] Rohden AI, Benchaya MC, Camargo RS, Moreira TC, Barros HMT, Ferigolo M (2017). Dropout Prevalence and Associated Factors in Randomized Clinical Trials of Adolescents Treated for Depression: Systematic Review and Meta-analysis. Clin Ther..

[34] DiFrancesco R, Rosenkranz SL, Craft J, Morse GD (2006). Tutorial reduces protocol deviations in multicenter ACTG trials with pharmacology endpoints. HIV Clin Trials..

[35] Nash JND (2007). Antidepressants. Psychiatry..

[36] Wiles N, Thomas L, Abel A, Ridgway N, Turner N, Campbell J (2013). Cognitive behavioural therapy as an adjunct to pharmacotherapy for primary care based patients with treatment resistant depression: results of the CoBalT randomised controlled trial. Lancet..

[37] Danielle T, Maud E, Jean-Yves FJPCH (2008). Adherence to treatment in adolescents..

[38] Administration UFaD. Suicidality in children and adolescents being treated with antidepressant medications. US Food and Drug Administration. Oct. 2004;15.

[39] McCulloch A, Kroll L, Glass J, Dubicka B. A systematic review of the characteristics of adolescents with major depressive disorder in randomised controlled treatment trials. The. Eur J Psychiatry. 2021.

[40] Cipriani A, Tomlinson A (2019). Providing the most appropriate care to our individual patients. Evid Based Ment Health..

