# Supplementary material for: Comparative short-term efficacy and acceptability of a combination of pharmacotherapy and psychotherapy for depressive disorder in children and adolescents: a systematic review and meta-analysis
Source: BMC Psychiatry. 2022 Feb 22;22:139. doi: 10.1186/s12888-022-03760-2 (PMC8862472; doi:10.1186/s12888-022-03760-2)

**Comparative short-term efficacy and acceptability of a combination of pharmacotherapy and psychotherapy for depressive disorder in children and adolescents: A systematic review and meta-analysis**

Yajie Xiang, Pim Cuijpers, Teng Teng, Xuemei Li, Li Fan, Xueer Liu, Yuanliang Jiang, Kang Du, Jingyuan Lin, Xinyu Zhou, Peng Xie

### **Supplementary appendix to the manuscript**

**Contents of Supplementary appendix**

| Appendix 1 | Search strategy and results | Page 3 |
| --- | --- | --- |
| Appendix 2 | Hierarchy of depression symptom severity measurement scales | Page 19 |
| Appendix 3 | Severity thresholds of rating scales | Page 21 |
| Appendix 4 | Subgroup analysis of acceptability for all studies | Page 23 |
| Appendix 5 | Sensitive analysis for each outcome | Page 25 |
| Appendix 6 | The summary of the ROB 2.0 assessment of each study | Page 27 |
| Appendix 7 | Funnel plot for each outcome from the meta-analysis | Page 29 |

**APPENDIX 1**

**Search strategy and result**

**Search Strategy and Results**

**Number of citations by each database and trial register searched***

| **Databases and Trial registers** | **Citations** |
| --- | --- |
| **Databases：** |  |
| Pubmed | 673 |
| Cochrane | 2189 |
| Web of Science | 423 |
| Embase | 564 |
| CINAHL | 320 |
| PsycINFO | 1125 |
| ProQuest Dissertations | 352 |
| LNACS | 21 |
| **Total (databases)** | **5667** |
|  |  |
| **Trial registers:** |  |
| USA (ClinicalTrials.gov) | 1523 |
| World Health Organization (ICTRP) | 1025 |
| USA Food and Drug Administration (FDA) | 1899 |
| Australian (ANZCTR) | 693 |
| China (ChiCTR) | 82 |
| Japan (IMIN-CTR) | 85 |
| Netherlands (Trial Register) | 25 |
| UN (ISRCTN) | 436 |
| **Total (trial registers)** | **5768** |

**Full search strategy for each database**

**PubMed**

#1 Search (depress*[Title/Abstract] OR dysthymi*[Title/Abstract] OR mood disorder*[Title/Abstract] OR affective disorder*[Title/Abstract])

#2 Search ((("Depressive Disorder"[Mesh]) OR "Dysthymic Disorder"[Mesh]) OR "Mood Disorders"[Mesh]) OR "Affective Disorders, Psychotic"[Mesh]

#3 #1 or #2

#4 Search (adolesc*[Title/Abstract] OR child*[Title/Abstract] OR boy*[Title/Abstract] OR girl*[Title/Abstract] OR juvenil*[Title/Abstract] OR minors[Title/Abstract] OR paediatri*[Title/Abstract] OR pediatri*[Title/Abstract] OR pubescen*[Title/Abstract] OR school*[Title/Abstract] OR student*[Title/Abstract] OR teen*[Title/Abstract] OR young[Title/Abstract] OR youth*[Title/Abstract] OR preschool[Title/Abstract] OR pre-school[Title/Abstract])

#5 Search "Child"[Majr] OR "Adolescent"[Majr]

#6 #4 or #5

#7 Search (antidepressant*[Title/Abstract] OR selective serotonin reuptake inhibitor*[Title/Abstract] OR SSRI[Title/Abstract] OR SSRIs[Title/Abstract] OR fluoxetine[Title/Abstract] OR fluvoxamine[Title/Abstract] OR paroxetine[Title/Abstract] OR sertraline[Title/Abstract] OR citalopram[Title/Abstract] OR escitalopram[Title/Abstract] OR vortioxetine[Title/Abstract] OR serotonin norepinephrine reuptake inhibitor*[Title/Abstract] OR SNRI[Title/Abstract] OR SNRIs[Title/Abstract] OR duloxetine[Title/Abstract] OR venlafaxine[Title/Abstract] OR desvenlafaxine[Title/Abstract] OR milnacipran[Title/Abstract] OR levomilnacipran[Title/Abstract] OR mirtazapine[Title/Abstract] OR mianserin[Title/Abstract] OR nefazodone[Title/Abstract] OR trazodone[Title/Abstract] OR vilazodone[Title/Abstract] OR bupropion[Title/Abstract] OR reboxetine[Title/Abstract] OR agomelatine[Title/Abstract] OR noradrenergic and specific serotonergic antidepressant*[Title/Abstract] OR NaSSA[Title/Abstract] OR NaSSAs[Title/Abstract] OR mirtazapine[Title/Abstract] OR TCA[Title/Abstract] OR TCAs[Title/Abstract] OR tricyclic[Title/Abstract] OR amersergide[Title/Abstract] OR amineptine[Title/Abstract] OR amitriptyline[Title/Abstract] OR amoxapine[Title/Abstract] OR butriptyline[Title/Abstract] OR chlorpoxiten[Title/Abstract] OR clomipramine[Title/Abstract] OR clorimipramine[Title/Abstract] OR demexiptiline[Title/Abstract] OR desipramine[Title/Abstract] OR dibenzipin[Title/Abstract] OR dothiepin[Title/Abstract] OR doxepin[Title/Abstract] OR imipramine[Title/Abstract] OR lofepramine[Title/Abstract] OR melitracen[Title/Abstract] OR metapramine[Title/Abstract] OR nortriptyline[Title/Abstract] OR noxiptiline[Title/Abstract] OR opipramol[Title/Abstract] OR protriptyline[Title/Abstract] OR quinupramine[Title/Abstract] OR tianeptine[Title/Abstract] OR trimipramine[Title/Abstract] OR “St John's Wort”[Title/Abstract] OR Hypericum perforatum[Title/Abstract] OR hypericin[Title/Abstract] OR hyperforin[Title/Abstract] OR johanniskraut[Title/Abstract])

#8 Search "Antidepressive Agents"[Mesh]

#9 #7 OR #8

#10 Search (psychother*[Title/Abstract] OR psychological[Title/Abstract] OR cogniti*[Title/Abstract] OR behavio*[Title/Abstract] OR CBT[Title/Abstract] OR family therap*[Title/Abstract] OR interpersonal[Title/Abstract] OR relaxation[Title/Abstract] OR bibliotherap*[Title/Abstract] OR play therap*[Title/Abstract] OR physical reinforcement[Title/Abstract] OR reinforced practice[Title/Abstract] OR exposure[Title/Abstract] OR consultation[Title/Abstract] OR biofeedback[Title/Abstract] OR social skill[Title/Abstract] OR client centered[Title/Abstract] OR counsel*[Title/Abstract] OR exercise[Title/Abstract] OR psychoeducation*[Title/Abstract] OR supportive[Title/Abstract] OR mental health[Title/Abstract] OR activity scheduling[Title/Abstract] OR art[Title/Abstract] OR dance[Title/Abstract] OR dialectic*[Title/Abstract] OR emotion focus*[Title/Abstract] OR focus-oriented[Title/Abstract] OR humanistic[Title/Abstract] OR integrative[Title/Abstract] OR integrated[Title/Abstract] OR metacognitive[Title/Abstract] OR meta-cognitive[Title/Abstract] OR nondirective[Title/Abstract] OR non-directive[Title/Abstract] OR problem solving[Title/Abstract] OR psychodynamic[Title/Abstract] OR rational emotive[Title/Abstract] OR self control*[Title/Abstract] OR self talk[Title/Abstract] OR self help[Title/Abstract] OR stress management[Title/Abstract] OR mind training[Title/Abstract] OR functional analys*[Title/Abstract] OR psychoanaly*[Title/Abstract] OR psychodrama[Title/Abstract] OR role play*[Title/Abstract] OR mindfulness*[Title/Abstract])

#11 Search "Psychotherapy"[Mesh]

#12 #10 OR #11

#13 #9 AND #12

#14 random* OR allocate*OR assign* OR “cross over*” OR crossover* OR controlled

#15 #3 AND #6 AND #13 #14

#16 Applied Filters: Humans

**Cochrane**

#1 depress* or dysthymi* or "mood disorder*" or "affective disorder*":ti or depress* or dysthymi* or "mood disorder*" or "affective disorder*":ab

#2 MeSH descriptor: [Depressive Disorder] explode all trees

#3 MeSH descriptor: [Dysthymic Disorder] explode all trees

#4 MeSH descriptor: [Mood Disorders] explode all trees

#5 #1 or #2 or #3 or #4

#6 adolesc* or child* or boy* or girl* or juvenil* or minors or paediatri* or pediatri* or pubescen* or school* or student* or teen* or young or youth* or preschool or pre-school:ti or adolesc* or child* or boy* or girl* or juvenil* or minors or paediatri* or pediatri* or pubescen* or school* or student* or teen* or young or youth* or preschool or pre-school:ab

#7 MeSH descriptor: [Child] explode all trees

#8 MeSH descriptor: [Adolescent] explode all trees

#9 #6 or #7 or #8

#10 antidepressant* or "selective serotonin reuptake inhibitor*" or SSRI or SSRIs or fluoxetine or fluvoxamine or paroxetine or sertraline or citalopram or escitalopram or vortioxetine or "serotonin norepinephrine reuptake inhibitor*" or SNRI or SNRIs or duloxetine or venlafaxine or desvenlafaxine or milnacipran or levomilnacipran or mirtazapine or mianserin or nefazodone or trazodone or vilazodone or bupropion or reboxetine or agomelatine or "noradrenergic and specific serotonergic antidepressant*" or NaSSA or NaSSAs or mirtazapine or TCA or TCAs or tricyclic or amersergide or amineptine or amitriptyline or amoxapine or butriptyline or chlorpoxiten or clomipramine or clorimipramine or demexiptiline or desipramine or dibenzipin or dothiepin or doxepin or imipramine or lofepramine or melitracen or metapramine or nortriptyline or noxiptiline or opipramol or protriptyline or quinupramine or tianeptine or trimipramine or “St John's Wort” or hypericin or hyperforin or johanniskraut:ti or antidepressant* or "selective serotonin reuptake inhibitor*" or SSRI or SSRIs or fluoxetine or fluvoxamine or paroxetine or sertraline or citalopram or escitalopram or vortioxetine or "serotonin norepinephrine reuptake inhibitor*" or SNRI or SNRIs or duloxetine or venlafaxine or desvenlafaxine or milnacipran or levomilnacipran or mirtazapine or mianserin or nefazodone or trazodone or vilazodone or bupropion or reboxetine or agomelatine or "noradrenergic and specific serotonergic antidepressant*" or NaSSA or NaSSAs or mirtazapine or TCA or TCAs or tricyclic or amersergide or amineptine or amitriptyline or amoxapine or butriptyline or chlorpoxiten or clomipramine or clorimipramine or demexiptiline or desipramine or dibenzipin or dothiepin or doxepin or imipramine or lofepramine or melitracen or metapramine or nortriptyline or noxiptiline or opipramol or protriptyline or quinupramine or tianeptine or trimipramine or “St John's Wort” or hypericin or hyperforin or johanniskraut:ab

#11 MeSH descriptor: [Antidepressive Agents] explode all trees

#12 #10 or #11

#13 psychother* or psychological or cogniti* or behavio* or CBT or "family therap*" or interpersonal or relaxation or bibliotherap* or "play therap*" or "physical reinforcement" or "reinforced practice" or exposure or consultation or biofeedback or "social skill" or "client centered" or counsel* or exercise or psychoeducation* or supportive or "mental health" or "activity scheduling" or art or dance or dialectic* or "emotion focus*" or "focus-oriented" or humanistic or integrative or integrated or metacognitive or meta-cognitive or nondirective or non-directive or "problem solving" or psychodynamic or "rational emotive" or "self control*" or "self talk" or "self help" or "stress management" or "mind training" or "functional analys*" or psychoanaly* or psychodrama or "role play*" or mindfulness*:ti or psychother* or psychological or cogniti* or behavio* or CBT or "family therap*" or interpersonal or relaxation or bibliotherap* or "play therap*" or "physical reinforcement" or "reinforced practice" or exposure or consultation or biofeedback or "social skill" or "client centered" or counsel* or exercise or psychoeducation* or supportive or "mental health" or "activity scheduling" or art or dance or dialectic* or "emotion focus*" or "focus-oriented" or humanistic or integrative or integrated or metacognitive or meta-cognitive or nondirective or non-directive or "problem solving" or psychodynamic or "rational emotive" or "self control*" or "self talk" or "self help" or "stress management" or "mind training" or "functional analys*" or psychoanaly* or psychodrama or "role play*" or mindfulness*:ab

#14 acceptance* or commitment*:ti or acceptance* or commitment*:ab

#15 *therap*:ti or *therap*:ab

#16 #14 and #15

#17 MeSH descriptor: [Psychotherapy] explode all trees

#18 #13 or #16 or #17

#19 #12 and #18

#20 (random* or allocate* or assign* or cross over* or crossover* or controlled):ti or (random* or allocate* or assign* or cross over* or crossover* or controlled):ab,kw

#21 #5 and #9 and #19 and #20

**Web of science**

#1 TS=(depress* or dysthymi* or “mood disorder*” or “affective disorder*”)

#2 TS=(adolesc* OR child* OR boy* OR girl* OR juvenil* OR minors OR paediatri* OR pediatri* OR pubescen* OR school* OR student* OR teen* OR young OR youth* OR preschool OR pre-school)

#3 TS=(antidepressant* or “selective serotonin reuptake inhibitor*” or SSRI or SSRIs or fluoxetine or fluvoxamine or paroxetine or sertraline or citalopram or escitalopram or vortioxetineor “serotonin norepinephrine reuptake inhibitor*” or SNRI or SNRIs or duloxetine or venlafaxine or desvenlafaxine or milnacipran or levomilnacipran or mirtazapine or mianserin or nefazodone or trazodone or vilazodone or bupropion or reboxetine or agomelatine or “noradrenergic and specific serotonergic antidepressant*” or NaSSA or NaSSAs or mirtazapine or TCA or TCAs or tricyclic or amersergide or amineptine or amitriptyline or amoxapine or butriptyline or chlorpoxiten or clomipramine or clorimipramine or demexiptiline or desipramine or dibenzipin or dothiepin or doxepin or imipramine or lofepramine or melitracen or metapramine or nortriptyline or noxiptiline or opipramol or protriptyline or quinupramine or tianeptine or trimipramine or St John's Wort or hypericin or hyperforin or johanniskraut)

#4 TS=(*therap* and (acceptance* or commitment*))

#5 TS=(psychother* or psychological or cogniti* or behavio* or CBT or “family therap*” or interpersonal or relaxation or bibliotherap* or “play therap*” or “physical reinforcement” or “reinforced practice” or exposure or consultation or biofeedback or “social skill” or “client centered” or counsel* or exercise or psychoeducation* or supportive or “mental health” or “activity scheduling” or art or dance or dialectic* or “emotion focus*” or “focus-oriented” or humanistic or integrative or integrated or metacognitive or meta-cognitive or nondirective or non-directive or “problem solving” or psychodynamic or “rational emotive” or “self control*” or “self talk” or “self help” or “stress management” or “mind training” or “functional analys*” or psychoanaly* or psychodrama or “role play*” or mindfulness*)

#6 #5 OR #4

#7 TS=(random* or allocate* or assign* or cross over* or crossover* or controlled)

#8 #6 AND #3

#9 #8 AND #7 AND #2 AND #1

#10 Applied Filters: ( ARTICLE ) AND Web of Science : ( PSYCHIATRY )

**Embase**

#1 depress*:ab,ti OR dysthymi*:ab,ti OR 'mood disorder*':ab,ti OR 'affective disorder*':ab,ti

#2 'depression'/exp OR 'dysthymia'/exp OR 'mood disorder'/exp

#3 #1 OR #2

#4 adolesc*:ab,ti OR child*:ab,ti OR boy*:ab,ti OR girl*:ab,ti OR juvenil*:ab,ti OR minors:ab,ti OR paediatri*:ab,ti OR pediatri*:ab,ti OR pubescen*:ab,ti OR school*:ab,ti OR student*:ab,ti OR teen*:ab,ti OR young:ab,ti OR youth*:ab,ti OR preschool:ab,ti OR 'preschool':ab,ti

#5 'child'/mj OR 'adolescent'/mj

#6 #4 OR #5

#7 antidepressant*:ab,ti OR 'selective serotonin reuptake inhibitor*':ab,ti OR ssri:ab,ti OR ssris:ab,ti OR fluoxetine:ab,ti OR fluvoxamine:ab,ti OR paroxetine:ab,ti OR sertraline:ab,ti OR citalopram:ab,ti OR escitalopram:ab,ti OR vortioxetine:ab,tiOR 'serotonin norepinephrine reuptake inhibitor*':ab,ti OR snri:ab,ti OR snris:ab,ti OR duloxetine:ab,ti OR venlafaxine:ab,ti OR desvenlafaxine:ab,ti OR milnacipran:ab,ti OR levomilnacipran:ab,ti OR mianserin:ab,ti OR nefazodone:ab,ti OR trazodone:ab,ti OR vilazodone:ab,ti OR bupropion:ab,ti OR reboxetine:ab,ti OR agomelatine:ab,ti OR 'noradrenergic and specific serotonergic antidepressant*':ab,ti OR nassa:ab,ti OR nassas:ab,ti OR mirtazapine:ab,ti OR tca:ab,ti OR tcas:ab,ti OR tricyclic:ab,ti OR amersergide:ab,ti OR amineptine:ab,ti OR amitriptyline:ab,ti OR amoxapine:ab,ti OR butriptyline:ab,ti OR chlorpoxiten:ab,ti OR clomipramine:ab,ti OR clorimipramine:ab,ti OR demexiptiline:ab,ti OR desipramine:ab,ti OR dibenzipin:ab,ti OR dothiepin:ab,ti OR doxepin:ab,ti OR imipramine:ab,ti OR lofepramine:ab,ti OR melitracen:ab,ti OR metapramine:ab,ti OR nortriptyline:ab,ti OR noxiptiline:ab,ti OR opipramol:ab,ti OR protriptyline:ab,ti OR quinupramine:ab,ti OR tianeptine:ab,ti OR trimipramine OR 'St John's Wort':ab,ti OR hypericin:ab,ti OR hyperforin:ab,ti OR johanniskraut:ab,ti

#8 'antidepressant agent'/exp

#9 #7 OR #8

#10 psychother*:ab,ti OR psychological:ab,ti OR cogniti*:ab,ti OR behavio*:ab,ti OR cbt:ab,ti OR 'family therap*':ab,ti OR interpersonal:ab,ti OR relaxation:ab,ti OR bibliotherap*:ab,ti OR 'play therap*':ab,ti OR 'physical reinforcement':ab,ti OR 'reinforced practice':ab,ti OR exposure:ab,ti OR consultation:ab,ti OR biofeedback:ab,ti OR 'social skill':ab,ti OR 'client centered':ab,ti OR counsel*:ab,ti OR exercise:ab,ti OR psychoeducation*:ab,ti OR supportive:ab,ti OR 'mental health':ab,ti OR 'activity scheduling':ab,ti OR art:ab,ti OR dance:ab,ti OR dialectic*:ab,ti OR 'emotion focus*':ab,ti OR 'focus-oriented':ab,ti OR humanistic:ab,ti OR integrative:ab,ti OR integrated:ab,ti OR metacognitive:ab,ti OR 'meta cognitive':ab,ti OR nondirective:ab,ti OR 'non directive':ab,ti OR 'problem solving':ab,ti OR psychodynamic:ab,ti OR 'rational emotive':ab,ti OR 'self control*':ab,ti OR 'self talk':ab,ti OR 'self help':ab,ti OR 'stress management':ab,ti OR 'mind training':ab,ti OR 'functional analys*':ab,ti OR psychoanaly*:ab,ti OR psychodrama:ab,ti OR 'role play*':ab,ti OR mindfulness*:ab,ti

#11 therap*:ab,ti AND (acceptance*:ab,ti OR commitment*:ab,ti)

#12 'psychotherapy'/exp

#13 #10 OR #11 OR #12

#14 #9 AND #13

#15 random*:ab,ti or allocate*:ab,ti or assign*:ab,ti or cross over*:ab,ti or crossover*:ab,ti or controlled:ab,ti

#16 #3 and #6 and #14 and #15

#17 Applied Filters: Humans and Article

**CINAHL**

S1 TI (depress* or dysthymi* or “mood disorder*” or “affective disorder*”) OR AB (depress* or dysthymi* or “mood disorder*” or “affective disorder*”)

S2 (MH "Affective Disorders+") OR (MH "Depression+") OR (MH "Dysthymic Disorder")

S3 S1 OR S2

S4 TI (adolesc* OR child* OR boy* OR girl* OR juvenil* OR minors OR paediatri* OR pediatri* OR pubescen* OR school* OR student* OR teen* OR young OR youth* OR preschool OR pre-school) OR AB (adolesc* OR child* OR boy* OR girl* OR juvenil* OR minors OR paediatri* OR pediatri* OR pubescen* OR school* OR student* OR teen* OR young OR youth* OR preschool OR pre-school)

S5 (MH "Child") OR (MH "Adolescence")

S6 S4 OR S5

S7 TI (antidepressant* or “selective serotonin reuptake inhibitor*” or SSRI or SSRIs or fluoxetine or fluvoxamine or paroxetine or sertraline or citalopram or escitalopram orvortioxetine or “serotonin norepinephrine reuptake inhibitor*” or SNRI or SNRIs or duloxetine or venlafaxine or desvenlafaxine or milnacipran or levomilnacipran or mirtazapine or mianserin or nefazodone or trazodone or vilazodone or bupropion or reboxetine or agomelatine or “noradrenergic and specific serotonergic antidepressant*” or NaSSA or NaSSAs or mirtazapine or TCA or TCAs or tricyclic or amersergide or amineptine or amitriptyline or amoxapine or butriptyline or chlorpoxiten or clomipramine or clorimipramine or demexiptiline or desipramine or dibenzipin or dothiepin or doxepin or imipramine or lofepramine or melitracen or metapramine or nortriptyline or noxiptiline or opipramol or protriptyline or quinupramine or tianeptine or trimipramine or St John's Wort or hypericin or hyperforin or johanniskraut) OR AB (antidepressant* or “selective serotonin reuptake inhibitor*” or SSRI or SSRIs or fluoxetine or fluvoxamine or paroxetine or sertraline or citalopram or escitalopram or “serotonin norepinephrine reuptake inhibitor*” or SNRI or SNRIs or duloxetine or venlafaxine or desvenlafaxine or milnacipran or levomilnacipran or mirtazapine or mianserin or nefazodone or trazodone or vilazodone or bupropion or reboxetine or agomelatine or “noradrenergic and specific serotonergic antidepressant*” or NaSSA or NaSSAs or mirtazapine or TCA or TCAs or tricyclic or amersergide or amineptine or amitriptyline or amoxapine or butriptyline or chlorpoxiten or clomipramine or clorimipramine or demexiptiline or desipramine or dibenzipin or dothiepin or doxepin or imipramine or lofepramine or melitracen or metapramine or nortriptyline or noxiptiline or opipramol or protriptyline or quinupramine or tianeptine or trimipramine or St John's Wort or hypericin or hyperforin or johanniskraut)

S8 (MH "Antidepressive Agents+")

S9 S7 OR S8

S10 TI (psychother* or psychological or cogniti* or behavio* or CBT or “family therap*” or interpersonal or relaxation or bibliotherap* or “play therap*” or “physical reinforcement” or “reinforced practice” or exposure or consultation or biofeedback or “social skill” or “client centered” or counsel* or exercise or psychoeducation* or supportive or “mental health” or “activity scheduling” or art or dance or dialectic* or “emotion focus*” or “focus-oriented” or humanistic or integrative or integrated or metacognitive or meta-cognitive or nondirective or non-directive or “problem solving” or psychodynamic or “rational emotive” or “self control*” or “self talk” or “self help” or “stress management” or “mind training” or “functional analys*” or psychoanaly* or psychodrama or “role play*” or mindfulness*) OR AB (psychother* or psychological or cogniti* or behavio* or CBT or “family therap*” or interpersonal or relaxation or bibliotherap* or “play therap*” or “physical reinforcement” or “reinforced practice” or exposure or consultation or biofeedback or “social skill” or “client centered” or counsel* or exercise or psychoeducation* or supportive or “mental health” or “activity scheduling” or art or dance or dialectic* or “emotion focus*” or “focus-oriented” or humanistic or integrative or integrated or metacognitive or meta-cognitive or nondirective or non-directive or “problem solving” or psychodynamic or “rational emotive” or “self control*” or “self talk” or “self help” or “stress management” or “mind training” or “functional analys*” or psychoanaly* or psychodrama or “role play*” or mindfulness*)

S11 TI *therap* OR AB *therap*

S12 TI (acceptance* or commitment*) OR AB (acceptance* or commitment*)

S13 S11 AND S12

S14 (MH "Psychotherapy+")

S15 S10 OR S13 OR S14

S16 TI (random* or allocate* or assign* or cross over* or crossover* or controlled) OR AB (random* or allocate* or assign* or cross over* or crossover* or controlled)

S17 (MH "Clinical Trials+")

S18 S16 OR S17

S19 S9 AND S15

S20 S3 AND S6 AND S18 AND S19

**PsycINFO**

S1 TI (depress* or dysthymi* or mood disorder* or affective disorder*) OR AB (depress* or dysthymi* or mood disorder* or affective disorder*)

S2 MA major depression OR MA affective disorders OR MA dysthymic disorder

S3 S1 OR S2

S4 TI (adolesc* or child* or boy* or girl* or juvenil* or minors or paediatri* or pediatri* or pubescen* or school* or student* or teen* or young or youth* or preschool or pre-school) OR AB (adolesc* or child* or boy* or girl* or juvenil* or minors or paediatri* or pediatri* or pubescen* or school* or student* or teen* or young or youth* or preschool or pre-school)

S5 MA child psychiatry

S6 MA adolescent psychiatry

S7 S4 OR S5 OR S6

S8 TI (antidepressant* or "selective serotonin reuptake inhibitor*" or SSRI or SSRIs or fluoxetine or fluvoxamine or paroxetine or sertraline or citalopram or escitalopram or vortioxetine or "serotonin norepinephrine reuptake inhibitor*" or SNRI or SNRIs or duloxetine or venlafaxine or desvenlafaxine or milnacipran or levomilnacipran or mirtazapine or mianserin or nefazodone or trazodone or vilazodone or bupropion or reboxetine or agomelatine or "noradrenergic and specific serotonergic antidepressant*" or NaSSA or NaSSAs or mirtazapine or TCA or TCAs or tricyclic or amersergide or amineptine or amitriptyline or amoxapine or butriptyline or chlorpoxiten or clomipramine or clorimipramine or demexiptiline or desipramine or dibenzipin or dothiepin or doxepin or imipramine or lofepramine or melitracen or metapramine or nortriptyline or noxiptiline or opipramol or protriptyline or quinupramine or tianeptine or trimipramine or St John's Wort or hypericin or hyperforin or johanniskraut) OR AB (antidepressant* or "selective serotonin reuptake inhibitor*" or SSRI or SSRIs or fluoxetine or fluvoxamine or paroxetine or sertraline or citalopram or escitalopram or vortioxetine or "serotonin norepinephrine reuptake inhibitor*" or SNRI or SNRIs or duloxetine or venlafaxine or desvenlafaxine or milnacipran or levomilnacipran or mirtazapine or mianserin or nefazodone or trazodone or vilazodone or bupropion or reboxetine or agomelatine or "noradrenergic and specific serotonergic antidepressant*" or NaSSA or NaSSAs or mirtazapine or TCA or TCAs or tricyclic or amersergide or amineptine or amitriptyline or amoxapine or butriptyline or chlorpoxiten or clomipramine or clorimipramine or demexiptiline or desipramine or dibenzipin or dothiepin or doxepin or imipramine or lofepramine or melitracen or metapramine or nortriptyline or noxiptiline or opipramol or protriptyline or quinupramine or tianeptine or trimipramine or St John's Wort or hypericin or hyperforin or johanniskraut)

S9 TI (psychother* or psychological or cogniti* or behavio* or CBT or "family therap*" or interpersonal or relaxation or bibliotherap* or "play therap*" or "physical reinforcement" or "reinforced practice" or exposure or consultation or biofeedback or "social skill" or "client centered" or counsel* or exercise or psychoeducation* or supportive or "mental health" or "activity scheduling" or art or dance or dialectic* or "emotion focus*" or "focus-oriented" or humanistic or integrative or integrated or metacognitive or meta-cognitive or nondirective or non-directive or "problem solving" or psychodynamic or "rational emotive" or "self control*" or "self talk" or "self help" or "stress management" or "mind training" or "functional analys*" or psychoanaly* or psychodrama or "role play*" or mindfulness*:ti or psychother* or psychological or cogniti* or behavio* or CBT or "family therap*" or interpersonal or relaxation or bibliotherap* or "play therap*" or "physical reinforcement" or "reinforced practice" or exposure or consultation or biofeedback or "social skill" or "client centered" or counsel* or exercise or psychoeducation* or supportive or "mental health" or "activity scheduling" or art or dance or dialectic* or "emotion focus*" or "focus-oriented" or humanistic or integrative or integrated or metacognitive or meta-cognitive or nondirective or non-directive or "problem solving" or psychodynamic or "rational emotive" or "self control*" or "self talk" or "self help" or "stress management" or "mind training" or "functional analys*" or psychoanaly* or psychodrama or "role play*" or mindfulness*) OR AB (psychother* or psychological or cogniti* or behavio* or CBT or "family therap*" or interpersonal or relaxation or bibliotherap* or "play therap*" or "physical reinforcement" or "reinforced practice" or exposure or consultation or biofeedback or "social skill" or "client centered" or counsel* or exercise or psychoeducation* or supportive or "mental health" or "activity scheduling" or art or dance or dialectic* or "emotion focus*" or "focus-oriented" or humanistic or integrative or integrated or metacognitive or meta-cognitive or nondirective or non-directive or "problem solving" or psychodynamic or "rational emotive" or "self control*" or "self talk" or "self help" or "stress management" or "mind training" or "functional analys*" or psychoanaly* or psychodrama or "role play*" or mindfulness*:ti or psychother* or psychological or cogniti* or behavio* or CBT or "family therap*" or interpersonal or relaxation or bibliotherap* or "play therap*" or "physical reinforcement" or "reinforced practice" or exposure or consultation or biofeedback or "social skill" or "client centered" or counsel* or exercise or psychoeducation* or supportive or "mental health" or "activity scheduling" or art or dance or dialectic* or "emotion focus*" or "focus-oriented" or humanistic or integrative or integrated or metacognitive or meta-cognitive or nondirective or non-directive or "problem solving" or psychodynamic or "rational emotive" or "self control*" or "self talk" or "self help" or "stress management" or "mind training" or "functional analys*" or psychoanaly* or psychodrama or "role play*" or mindfulness*)

S10 TI (acceptance* or commitment*) OR AB (acceptance* or commitment*)

S11 TI therap* OR AB therap*

S12 S10 and S11

S13 S9 or S12

S14 exp psychotherapy/

S15 S13 or S14

S16 exp clinical trials/

S17 (random* or allocate* or assign* or cross over* or crossover* or controlled) OR AB (random* or allocate* or assign* or cross over* or crossover* or controlled).ab

S18 S16 or S17

S19 S8 and S15

S20 S3 and S7 and S18 and S19

**ProQuest Dissertations**

S1 [ti(depress* or dysthymi* or “mood disorder*” or “affective disorder*”) OR ab(depress* or dysthymi* or “mood disorder*” or “affective disorder*”) OR su("Affective Disorders*" or depress* or "Dysthymic Disorder")](http://search.proquest.com.er.lib.ncku.edu.tw:2048/recentsearches.recentsearchtabview.recentsearchesgridview.scrolledrecentsearchlist.checkdbssearchlink:rerunsearch/665678E6D4AA4D2FPQ/None?site=pqdt&t:ac=RecentSearches)

S2 [ti(adolesc* OR child* OR boy* OR girl* OR juvenil* OR minors OR paediatri* OR pediatri* OR pubescen* OR school* OR student* OR teen* OR young OR youth* OR preschool OR pre-school) OR ab(adolesc* OR child* OR boy* OR girl* OR juvenil* OR minors OR paediatri* OR pediatri* OR pubescen* OR school* OR student* OR teen* OR young OR youth* OR preschool OR pre-school) OR su(Child or Adolescence)](http://search.proquest.com.er.lib.ncku.edu.tw:2048/recentsearches.recentsearchtabview.recentsearchesgridview.scrolledrecentsearchlist.checkdbssearchlink:rerunsearch/2E85DDFF441A4B27PQ/None?site=pqdt&t:ac=RecentSearches)

S3 ti(antidepressant* or "selective serotonin reuptake inhibitor*" or SSRI or SSRIs or fluoxetine or fluvoxamine or paroxetine or sertraline or citalopram or escitalopram or vortioxetine or "serotonin norepinephrine reuptake inhibitor*" or SNRI or SNRIs or duloxetine or venlafaxine or desvenlafaxine or milnacipran or levomilnacipran or mirtazapine or mianserin or nefazodone or trazodone or vilazodone or bupropion or reboxetine or agomelatine or "noradrenergic and specific serotonergic antidepressant*" or NaSSA or NaSSAs or mirtazapine or TCA or TCAs or tricyclic or amersergide or amineptine or amitriptyline or amoxapine or butriptyline or chlorpoxiten or clomipramine or clorimipramine or demexiptiline or desipramine or dibenzipin or dothiepin or doxepin or imipramine or lofepramine or melitracen or metapramine or nortriptyline or noxiptiline or opipramol or protriptyline or quinupramine or tianeptine or trimipramine or St John's Wort or hypericin or hyperforin or johanniskraut) OR ab(antidepressant* or "selective serotonin reuptake inhibitor*" or SSRI or SSRIs or fluoxetine or fluvoxamine or paroxetine or sertraline or citalopram or escitalopram or vortioxetine or "serotonin norepinephrine reuptake inhibitor*" or SNRI or SNRIs or duloxetine or venlafaxine or desvenlafaxine or milnacipran or levomilnacipran or mirtazapine or mianserin or nefazodone or trazodone or vilazodone or bupropion or reboxetine or agomelatine or "noradrenergic and specific serotonergic antidepressant*" or NaSSA or NaSSAs or mirtazapine or TCA or TCAs or tricyclic or amersergide or amineptine or amitriptyline or amoxapine or butriptyline or chlorpoxiten or clomipramine or clorimipramine or demexiptiline or desipramine or dibenzipin or dothiepin or doxepin or imipramine or lofepramine or melitracen or metapramine or nortriptyline or noxiptiline or opipramol or protriptyline or quinupramine or tianeptine or trimipramine or St John's Wort or hypericin or hyperforin or johanniskraut)

S4 ti(psychother* or psychological or cogniti* or behavio* or CBT or "family therap*" or interpersonal or relaxation or bibliotherap* or "play therap*" or "physical reinforcement" or "reinforced practice" or exposure or consultation or biofeedback or "social skill" or "client centered" or counsel* or exercise or psychoeducation* or supportive or "mental health" or "activity scheduling" or art or dance or dialectic* or "emotion focus*" or "focus-oriented" or humanistic or integrative or integrated or metacognitive or meta-cognitive or nondirective or non-directive or "problem solving" or psychodynamic or "rational emotive" or "self control*" or "self talk" or "self help" or "stress management" or "mind training" or "functional analys*" or psychoanaly* or psychodrama or "role play*" or mindfulness*:ti or psychother* or psychological or cogniti* or behavio* or CBT or "family therap*" or interpersonal or relaxation or bibliotherap* or "play therap*" or "physical reinforcement" or "reinforced practice" or exposure or consultation or biofeedback or "social skill" or "client centered" or counsel* or exercise or psychoeducation* or supportive or "mental health" or "activity scheduling" or art or dance or dialectic* or "emotion focus*" or "focus-oriented" or humanistic or integrative or integrated or metacognitive or meta-cognitive or nondirective or non-directive or "problem solving" or psychodynamic or "rational emotive" or "self control*" or "self talk" or "self help" or "stress management" or "mind training" or "functional analys*" or psychoanaly* or psychodrama or "role play*" or mindfulness*) OR ab(psychother* or psychological or cogniti* or behavio* or CBT or "family therap*" or interpersonal or relaxation or bibliotherap* or "play therap*" or "physical reinforcement" or "reinforced practice" or exposure or consultation or biofeedback or "social skill" or "client centered" or counsel* or exercise or psychoeducation* or supportive or "mental health" or "activity scheduling" or art or dance or dialectic* or "emotion focus*" or "focus-oriented" or humanistic or integrative or integrated or metacognitive or meta-cognitive or nondirective or non-directive or "problem solving" or psychodynamic or "rational emotive" or "self control*" or "self talk" or "self help" or "stress management" or "mind training" or "functional analys*" or psychoanaly* or psychodrama or "role play*" or mindfulness*:ti or psychother* or psychological or cogniti* or behavio* or CBT or "family therap*" or interpersonal or relaxation or bibliotherap* or "play therap*" or "physical reinforcement" or "reinforced practice" or exposure or consultation or biofeedback or "social skill" or "client centered" or counsel* or exercise or psychoeducation* or supportive or "mental health" or "activity scheduling" or art or dance or dialectic* or "emotion focus*" or "focus-oriented" or humanistic or integrative or integrated or metacognitive or meta-cognitive or nondirective or non-directive or "problem solving" or psychodynamic or "rational emotive" or "self control*" or "self talk" or "self help" or "stress management" or "mind training" or "functional analys*" or psychoanaly* or psychodrama or "role play*" or mindfulness*)

S5 ti(therap*) OR ab( therap*)

S6 ti(acceptance* OR commitment*) OR ab(acceptance* OR commitment*)

S7 S5 AND S6

S8 S4 OR S7

S9 S3 AND S8

S10 ti(random* or allocate* or assign* or cross over* or crossover* or controlled) or ab(random* or allocate* or assign* or cross over* or crossover* or controlled)

S11 S1 AND S2 AND S9 AND S11

**LILACS**

S1 (depress$ or dysthymi$ or “mood disorder$” or “affective disorder$”) and (adolesc$ OR child$ OR boy$ OR girl$ OR juvenil$ OR minors OR paediatri$ OR pediatri$ OR pubescen$ OR school$ OR student$ OR teen$ OR young OR youth$ OR preschool OR pre-school) [Words] and (antidepressant$ or "selective serotonin reuptake inhibitor$" or SSRI or SSRIs or fluoxetine or fluvoxamine or paroxetine or sertraline or citalopram or escitalopram or vortioxetine or "serotonin norepinephrine reuptake inhibitor$" or SNRI or SNRIs or duloxetine or venlafaxine or desvenlafaxine or milnacipran or levomilnacipran or mirtazapine or mianserin or nefazodone or trazodone or vilazodone or bupropion or reboxetine or agomelatine or "noradrenergic and specific serotonergic antidepressant$" or NaSSA or NaSSAs or mirtazapine or TCA or TCAs or tricyclic or amersergide or amineptine or amitriptyline or amoxapine or butriptyline or chlorpoxiten or clomipramine or clorimipramine or demexiptiline or desipramine or dibenzipin or dothiepin or doxepin or imipramine or lofepramine or melitracen or metapramine or nortriptyline or noxiptiline or opipramol or protriptyline or quinupramine or tianeptine or trimipramine or St John's Wort or hypericin or hyperforin or johanniskraut) [Words] and (psychother$ or psychological or cogniti$ or behavio$ or CBT or "family therap$" or interpersonal or relaxation or bibliotherap$ or "play therap$" or "physical reinforcement" or "reinforced practice" or exposure or consultation or biofeedback or "social skill" or "client centered" or counsel$ or exercise or psychoeducation$ or supportive or "mental health" or "activity scheduling" or art or dance or dialectic$ or "emotion focus$" or "focus-oriented" or humanistic or integrative or integrated or metacognitive or meta-cognitive or nondirective or non-directive or "problem solving" or psychodynamic or "rational emotive" or "self control$" or "self talk" or "self help" or "stress management" or "mind training" or "functional analys$" or psychoanaly$ or psychodrama or "role play$" or mindfulness$:ti or psychother$ or psychological or cogniti$ or behavio$ or CBT or "family therap$" or interpersonal or relaxation or bibliotherap$ or "play therap$" or "physical reinforcement" or "reinforced practice" or exposure or consultation or biofeedback or "social skill" or "client centered" or counsel$ or exercise or psychoeducation$ or supportive or "mental health" or "activity scheduling" or art or dance or dialectic$ or "emotion focus$" or "focus-oriented" or humanistic or integrative or integrated or metacognitive or meta-cognitive or nondirective or non-directive or "problem solving" or psychodynamic or "rational emotive" or "self control$" or "self talk" or "self help" or "stress management" or "mind training" or "functional analys$" or psychoanaly$ or psychodrama or "role play$" or mindfulness$) [Words] and random$ or allocate$ or assign$ or cross over$ or crossover$ or controlled [Words]

**APPENDIX 2**

**Hierarchy of depression symptom severity measurement scales**

| **Hierarchy Depression symptom severity measurement scales and Abbreviations** | | |
| --- | --- | --- |
| 1 | Children’s Depression Rating Scale | CDRS |
| 2 | Hamilton Depression Rating Scale | HAMD |
| 3 | Montgomery Asberg Depression Rating Scale | MADRS |
| 4 | Beck Depression Inventory | BDI |
| 5 | Children’s Depression Inventory | CDI |
| 6 | Schedule for Affective Disorders and Schizophrenia for School Aged Children | K-SADS |
| 7 | Mood and Feeling Questionnaire | MFQ |
| 8 | Reynolds Adolescent Depression Scale | RADS |
| 9 | Bellevue Index of Depression | BID |
| 10 | Child Depression Scale | CDS |
| 11 | Centre for Epidemiologic Studies Depression Scale | CESD |
| 12 | Child Assessment Schedule | CAS |
| 13 | Child Behaviour Checklist-Depression | CBCL-D |

**APPENDIX 3**

**Severity thresholds of rating scales**

| **Rating scale** | **Studies** | **Severity thresholds** |
| --- | --- | --- |
| CDRS-R | Bernstein 2000, Fristad 2016, Goodyer 2008, Gunlicks-Stoessel 2019, Mandoki 1997, March 2004, Riggs 2007 | <45 = mild depression, 45-98 = moderate depression, and >98 = serve depression. |
| HAMD-14 | Clarke 2005 | No thresholds were available, we used the original reported severity from the study. |
| HAMD-24 | Deas 2000 | 0-8 = normal, 8-20 = mild depression, 20-35 = moderate depression, and >35 = serve depression. |
| HAMD-27 | Cornelius 2009 | No thresholds were available, we used the original reported severity from the study. |
| MADRS | Davey 2019 | 0-6 = normal, 7-19 = mild depression, 20-34 = moderate depression, and >34 = serve depression. |
| BDI | Kim 2012 | 0-13 = minimal depression, 14-19 = mild depression, 20-28 = moderate depression, and 29-63 = serve depression. |
| CDI-27 | Iftene F 2015 | 0-15 = normal, 15-20 = mild depression, 20-25 = moderate depression, and >25 = serve depression. |
| RADS | Melvin 2006 | 30-75 = normal, 76-81 = mild depression, 82-88 = moderate depression, and >89 = serve depression. |

CDRS-R = Children’s Depression Rating Scale-Revised. HAMD = Hamilton Rating Scale for Depression. MADRS = Montgomery-Asberg Depression Rating Scale. BDI = Beck Depression Inventory. CDI =Children’s Depression Inventory. RADS = Reynolds Adolescent Depression Scale.

**APPENDIX 4**

**Subgroup analysis of acceptability for all studies**

**Subgroup analysis of acceptability for all studies**

| **Variable** | **No of studies** | **Odds ratio (95% CI)** | **Chi^2^** | **I^2^** | ***P* value** | **Statistical power** |
| --- | --- | --- | --- | --- | --- | --- |
| **Type of other active treatment options** |  |  |  |  |  |  |
| Combined therapy vs pharmacotherapy | 7 | 1.05 (0.66 to 1.66) | 2.40 | 0% | 0.88 | 4.41% |
| Combined therapy vs psychotherapy | 4 | 0.85 (0.40 to 1.82) | 3.16 | 5% | 0.37 | 5.24% |
| Combined therapy vs Pill-PBO combined psychotherapy | 6 | 1.04 (0.55 to 1.96) | 3.84 | 0% | 0.57 | 4.49% |
| **Type of medication combined therapy** |  |  |  |  |  |  |
| Fluoxetine combined therapy | 5 | 0.79 (0.47 to 1.32) | 2.51 | 0% | 0.64 | 19.20% |
| Other SSRIs combined therapy | 7 | 1.38 (0.82 to 2.32) | 3.97 | 0% | 0.68 | 20.43% |
| Non-SSRIs combined therapy | 5 | 0.82 (0.39 to 1.76) | 0.75 | 0% | 0.95 | 5.06% |
| **Type of psychotherapy combined therapy** |  |  |  |  |  |  |
| CBT combined therapy | 14 | 1.01 (0.73 to 1.42) | 9.21 | 0% | 0.76 | 4.76% |
| Non-CBT combined therapy | 3 | 0.62 (0.12 to 3.20) | 0.19 | 0% | 0.91 | 3.54% |
| **Severity of baseline symptom** |  |  |  |  |  |  |
| Mild severity | 5 | 0.95 (0.30 to 2.98) | 3.81 | 0% | 0.43 | 4.04% |
| Moderate to server severity | 12 | 1.00 (0.71 to 1.41) | 5.93 | 0% | 0.88 | 4.71% |
| **Treatment duration** |  |  |  |  |  |  |
| Treatment ≤ 8 weeks | 7 | 0.98 (0.57 to 1.69) | 0.84 | 0% | 0.99 | 4.32% |
| Treatment > 8 weeks | 10 | 1.00 (0.66 to 1.52) | 8.88 | 0% | 0.45 | 5.57% |
| **Country** |  |  |  |  |  |  |
| USA | 11 | 0.90 (0.61 to 1.32) | 5.49 | 0% | 0.86 | 7.07% |
| Non-USA | 6 | 1.32 (0.70 to 2.50) | 3.20 | 0% | 0.67 | 7.10% |
| **Risk of bias** |  |  |  |  |  |  |
| Low risk | 3 | 1.50 (0.59 to 3.85) | 1.22 | 0% | 0.54 | 10.45% |
| Some concerns | 10 | 1.13 (0.65 to 1.95) | 5.88 | 0% | 0.75 | 5.57% |
| High risk | 4 | 0.83 (0.52 to 1.31) | 1.05 | 0% | 0.79 | 8.71% |

CI = confidence interval. *P* = level of significance of heterogeneity. I² = measure of heterogeneity.

Pill-PBO=Pill placebo. SSRIs= Selective serotonin reuptake inhibitors. CBT = Cognitive behavioural therapy. USA= United States of America.

**APPENDIX 5**

**Sensitivity analyses for each outcome**

**Summary of the sensitivity analyses for all outcomes**

| **Outcomes** | **All trials** | **Omitting non-blind trials** | **Omitting potential publication bias study** | **Omitting high risk trials of ROB 2.0** |
| --- | --- | --- | --- | --- |
| **Remission**  **Odds ratio (95% CI)** | 1.37 (0.93 to 2.04) | 1.44 (0.94 to 2.21) | 1.46 (0.98 to 2.18) | 0.98 (0.55 to 1.74) |
| **All-caused discontinuation**  **Odds ratio (95% CI)** | 0.99 (0.72 to 1.38) | 0.96 (0.69 to 1.35) | … | 1.21 (0.76 to 1.95) |
| **Efficacy at post-treatment**  **Standar mean difference (95% CI)** | -0.07 (-0.32 to 0.19) | -0.09 (-0.36 to 0.18) | 0.00 (-0.15 to 0.15) | 0.08 (-0.10 to 0.27) |
| **Suicidality**  **Odds ratio (95% CI)** | 1.17 (0.67 to 2.06) | 1.18 (0.65 to 2.13) | 1.34 (0.67 to 2.65) | 1.34 (0.67 to 2.65) |

**APPENDIX 6**

**The summary of the ROB 2.0 assessment of each study**

**The summary of the ROB 2.0 assessment of each study.**


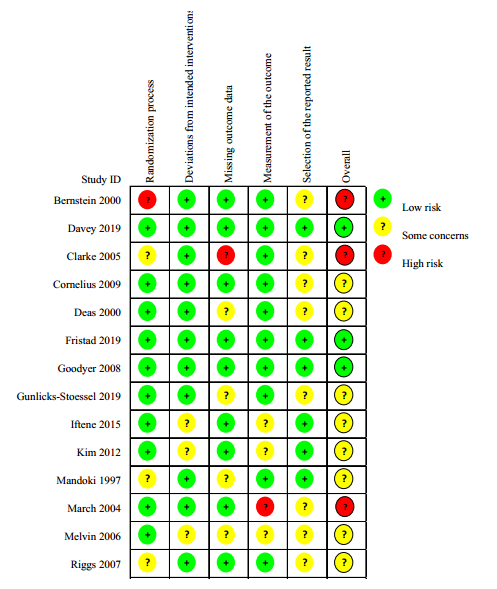


**APPENDIX 7**

**Funnel plot for each outcome from the meta-analysis**

The funnel plots of the meta-analysis weren’t suggestive of obvious publication bias for all outcomes. The value of Egger’s test is showed in the bottom of the funnel plot.

**a. Funnel plot forremission at post-treatment**


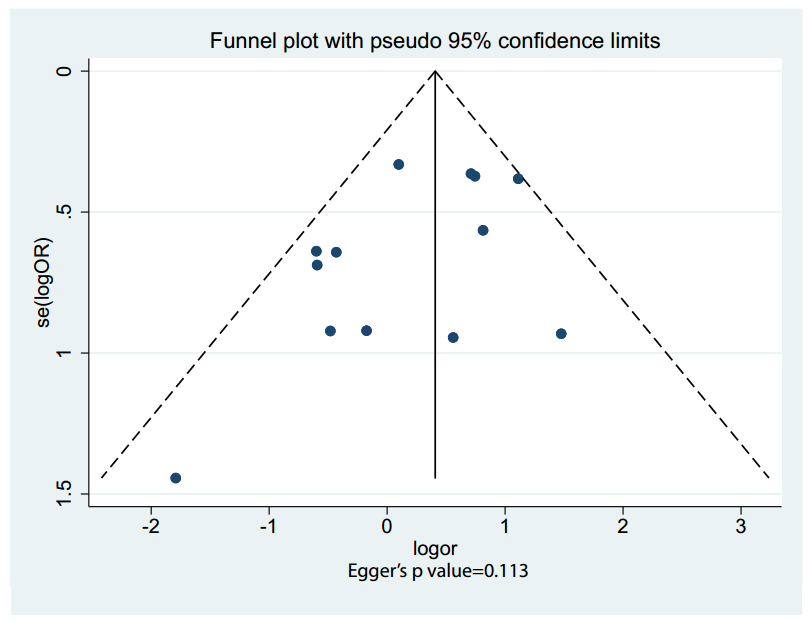


**b. Funnel plot for all-cause discontinuation**


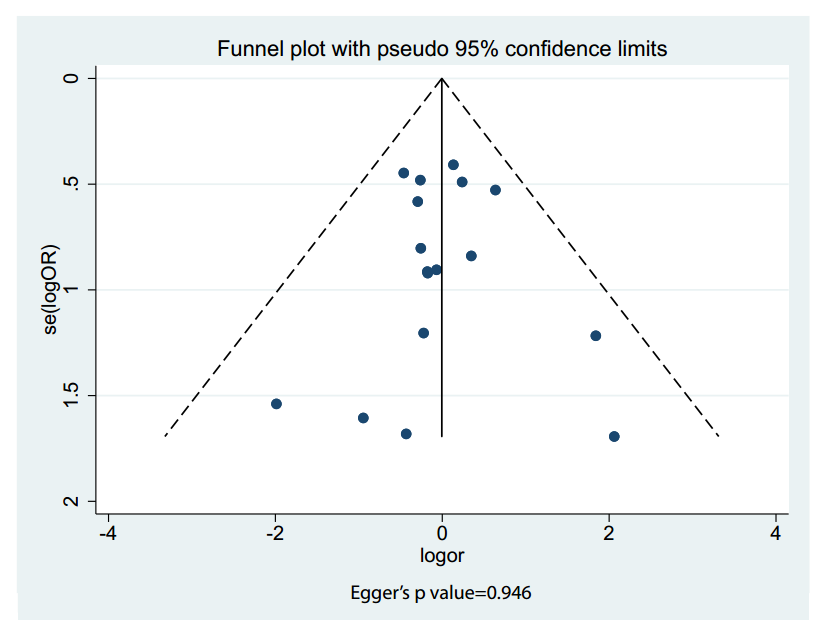


**c. Funnel plot for efficacy at post-treatment**


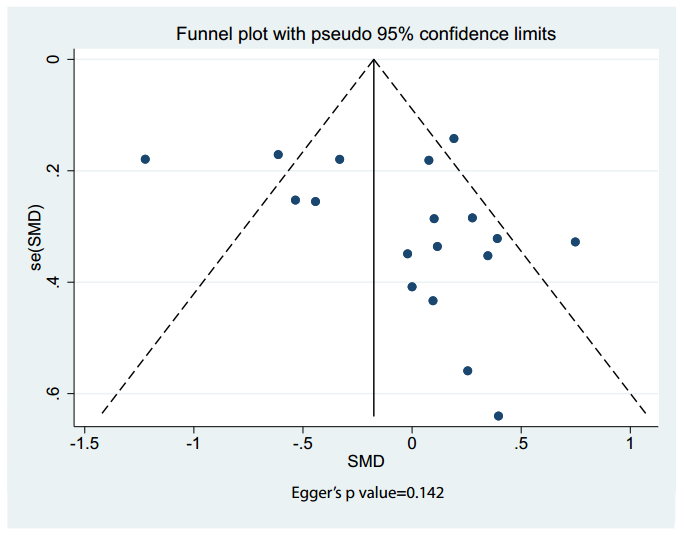


**d. Comparison-adjusted funnel plot for suicidality**


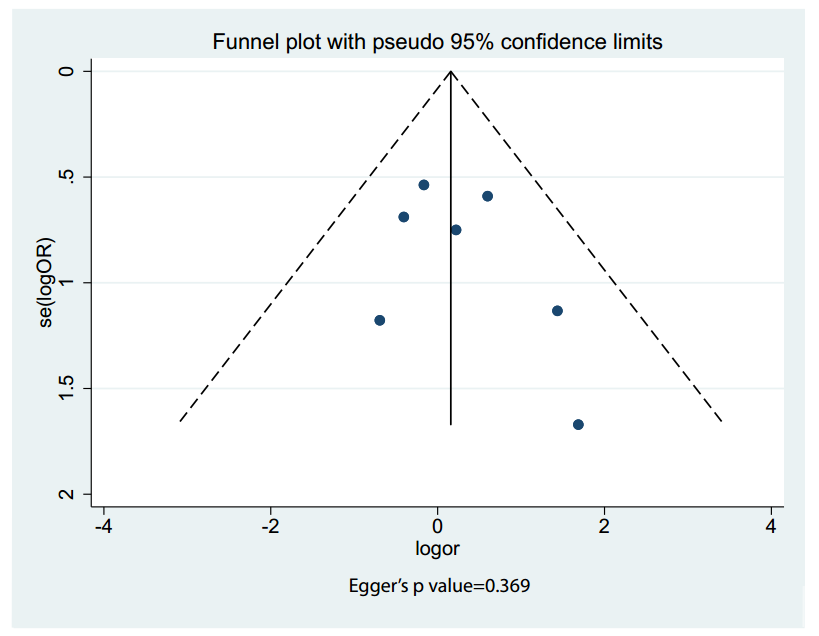

Supplement: Supplementary file 1 — Additional file 1. [file 12888_2022_3760_MOESM1_ESM.docx]
